# The component structure of the scales for the assessment of positive and negative symptoms in first-episode psychosis and its dependence on variations in analytic methods

**DOI:** 10.1016/j.psychres.2018.10.046

**Published:** 2018-12

**Authors:** Marc S. Tibber, James B. Kirkbride, Eileen M. Joyce, Stanley Mutsatsa, Isobel Harrison, Thomas R.E. Barnes, Vyv Huddy

**Affiliations:** a Department of Clinical, Educational and Health Psychology, UCL, London, UK; b Division of Psychiatry, UCL, London, UK; c UCL Institute of Neurology, London, UK; d School of Health Sciences, City University, London, UK; e Centre for Psychiatry, Imperial College London, UK; f Clinical Psychology Unit, Department of Psychology, University of Sheffield, UK

**Keywords:** Psychosis, Positive symptoms, Negative symptoms, Disorganisation symptoms, Factor analysis, Factor structure

## Abstract

A secondary analysis was undertaken on Scales for the Assessment of Positive and Negative Symptoms (SAPS/SANS) data from 345 first-episode psychosis (FEP) patients gathered in the West London FEP study. The purpose of this study was to determine: (i) the component structure of these measures in FEP (primary analyses), and (ii) the dependence of any findings in these primary analyses on variations in analytic methods. Symptom ratings were exposed to data reduction methods and the effects of the following manipulations ascertained: (i) level of analysis (individual symptom vs. global symptom severity ratings), (ii) extraction method (principal component vs. exploratory factor analysis) and (iii) retention method (scree test vs. Kaiser criterion). Whilst global ratings level analysis rendered the classic triad of psychotic syndromes (positive, negative and disorganisation), symptom level analyses revealed a hierarchical structure, with 11 first-order components subsumed by three second-order components, which also mapped on to this syndrome triad. These results were robust across data reduction but *not* component retention methods, suggesting that discrepancies in the literature regarding the component structure of the SAPS/SANS partly reflect the level of analysis and component retention method used. Further, they support a hierarchical symptom model, the implications of which are discussed.

## Highlights

• Psychotic symptoms show a complex hierarchical symptom structure.
• Ten+first-order factors were extracted using data reduction methods.
• Three second-order factors were extracted using data reduction methods.
• Some reported variance in symptom structure is due to analytic methods used.

## 1 Introduction

Psychotic disorders represent a broad family of psychological conditions that are characterised by cognitive, affective, perceptual, behavioural, and social symptoms (American Psychiatric Association 2013, World Health Organisation 1992). Whilst the psychoses have traditionally been described using a categorical approach based on diagnostic criteria (Parker, 2014, World Health Organization 1992), a number of authors have argued that psychotic disorders in fact represent a heterogeneous collection of phenomena (Allardyce et al., 2007a, Bentall, 2004), with diagnostic categories demonstrating poor validity, reliability and aetiological specificity. As an alternative dimensional based classification systems posit that psychosis may be better formulated with respect to multiple, continuous symptom dimensions (Allardyce et al., 2007b, Van Os, 2015). This is a view that has permeated both research and clinical practice. For example, the latest version of the Diagnostic and Statistical Manual of Mental Disorders (DSM-5) (American Psychiatric Association, 2013) carries a rating tool called the Clinician-Rated Dimensions of Psychosis Symptom Severity (Parker, 2014), and further, no longer includes the traditional sub-types of schizophrenia (e.g. paranoid, disorganised, catatonic etc.) on the basis that they lack reliability, validity and stability (Nemeroff et al., 2013). A comprehensive understanding of the underlying structure of psychotic symptoms is therefore critical.

Psychotic symptom dimensions are typically derived using the data reduction methods of exploratory factor analysis (EFA) or principal component analysis (PCA). However, there is great variability in the number of symptom dimensions identified using these techniques (Peralta and Cuesta, 2001), ranging from an early ten syndrome model (Lorr et al., 1963) to later proposals of a core triad of syndromes: reality distortion or ‘positive’ symptoms (certain delusions and hallucinations), psychomotor poverty or ‘negative’ symptoms (poverty of speech, lack of spontaneous movement and aspects of blunted affect) and disorganisation symptoms (inappropriate affect, poverty of content of speech and disturbances of the form of thought) (Liddle, 1987, Liddle and Barnes, 1990). See Grube et al., 1998, Peralta and Cuesta, 2001 and Smith et al. (1998) for reviews and indications of the variation in proposed symptom models.

A number of methodological issues that might contribute to this variability in symptom structure have been suggested (Peralta and Cuesta, 2001, Toomey et al., 1997) including: the characteristics of the patient sample, e.g. first-episode or established illness, the choice of symptom rating tool, the data reduction method, e.g. EFA or PCA, and the method used to determine the number of factors to extract, e.g. scree test or Kaiser criterion (eigenvector > 1) method. The choice of symptom rating tool may be particularly critical, since scales that incorporate a more comprehensive list of symptoms, such as the Positive and Negative Syndrome Scale (PANSS: van Erp et al., 2014) often generate a greater number of symptom dimensions (Peralta and Cuesta, 2001). For example, five factor models, which commonly include the classic triad of symptoms (positive, negative and disorganisation) as well as mania (/excitement/activation) and depression (/emotional distress) are also commonly reported; see Wallwork et al., 2012, Shafer et al., 2017 and van der Gaag et al. (2006) for example.

Another potential source of variation in the literature is the level of analysis undertaken, i.e. what constitutes the raw material for data reduction: individual symptom scores or scores on symptom sub-scales (or some other composite measure/index). This is particularly relevant for the Scales for the Assessment of Positive and Negative Symptoms (SAPS and SANS: Andreasen, 1990), which are commonly used for rating psychotic symptoms. The SAPS and SANS together are comprised of 49 individual symptom items, e.g. ‘auditory hallucinations’, in addition to nine sub-scale global symptom severity summary scores, e.g. ‘global rating of hallucinations’. Whilst individual symptom level analyses of SAPS and SANS ratings typically generate around ten factors (Minas et al., 1994, Peralta and Cuesta, 1999, Toomey et al., 1997, Vazquez-Barquero et al., 1996), global rating level analyses generate far fewer (typically three to four), including positive, negative and disorganisation syndromes (Dollfus and Petit, 1995, John et al., 2003, Klimidis et al., 1993, Liddle, 1987, Peralta and Cuesta, 1999, Toomey et al., 1997). Consequently, existing studies and reviews of the literature that include data from the SAPS and SANS (Grube et al., 1998, Smith et al., 1998) may underestimate the number of psychotic symptom dimensions as a result of the predominance of global level analyses (Stuart et al., 1999).

One study that has the potential to resolve some of this variation in the literature involved a symptom level PCA undertaken on SAPS and SANS ratings from 660 inpatients with psychotic illness (Peralta and Cuesta, 1999). This resulted in the extraction of 12 inter-correlated, first-order components. Critically however, the authors went on to use these first-order component scores as raw data for a second-order PCA; this resulted in four second-order components, three of which accurately mapped on to the positive, negative and disorganisation syndromes (Liddle, 1987, Liddle and Barnes, 1990). These findings suggest that psychotic symptoms may be inherently hierarchically structured, with ten or more symptom clusters (Lorr et al., 1963) defining a handful of higher-level clusters (or syndromes), including Liddle's classic triad.

Taken together, these findings suggest that discrepancies in the literature as to the dimensional structure of common psychotic symptom measures may be driven, in part, by variations in analytic method. Further, they suggest that some of the disparate findings reported might be integrated into a two-tiered hierarchical model (Peralta and Cuesta, 1999). To test this directly, we undertook a series of symptom level and global rating level analyses of SAPS and SANS scores in first-episode psychosis (FEP). Two primary hypotheses were tested: first, that the derived symptom structure would depend on the level of analysis undertaken. Specifically, we predicted that whilst a global ratings level analysis would lead to the extraction of the classic triad of syndromes, symptom level analysis would generate approximately ten first-order components. Second, we predicted, that in support of Peralta and Cuesta's (1999) hierarchical symptom model, it would be possible to recover the classic triad of syndromes by undertaking a second-order symptom level analysis. Finally, in order to explore how different statistical approaches may have shaped discrepancies in the literature as to the underlying structure of the SAPS and SANS, we also explored the dependence of any findings on common variations in method of data reduction or component retention (Peralta and Cuesta, 2001, Toomey et al., 1997).

## 2 Methods

### 2.1 Setting

The data were collected as part of the prospective West London First-Episode Psychosis study (WLFEP: Barnes et al., 2000, Joyce et al., 2005). Participants had presented to secondary care services within the London boroughs of Ealing, Hammersmith and Fulham, Wandsworth, Kingston, Richmond, Merton, Sutton and Hounslow, between 1998 and 2006. Ethical approval was obtained from local ethics committees of all boroughs included and written informed consent was obtained.

### 2.2 Participants

Patients were deemed eligible for inclusion in the study if they were resident in London (defined as any borough within the M25), aged 16 years or older, experiencing a first psychotic episode (affective or non-affective), had received fewer than 12 weeks of antipsychotic medication and had sufficient command of the English language to facilitate assessment. Potential participants were initially screened for a psychotic disorder using the World Health Organization Psychosis Screen (Jablensky et al., 1992). Where a psychotic disorder was indeed indicated, a full diagnosis was derived using a comprehensive structured interview known as the diagnostic module of the Diagnostic Interview for Psychosis (Castle et al., 2006), which includes items from the World Health Organization Schedules for Clinical Assessment in Neuropsychiatry (SCAN; Wing et al., 1990) and the Operational Criteria Checklist for Psychosis (OPCRIT; McGuffin et al., 1991). Information derived from this interview was then fed into a computer algorithm (MRC Social Genetic and Dev Psychiatry Centre, n.d.) to generate diagnoses according to multiple classification systems including the Diagnostic and Statistical Manual of Mental Disorders (3rd ed.; DSM-III; American Psychiatric Association, 1980) and subsequently converted into DSM-IV categories by cross-referencing with DSM-IV criteria (American Psychiatric Association, 1994). These screening and diagnostic assessment stages were undertaken by two psychiatric research nurses (IH and SM) trained in the administration of relevant tools by a highly experienced psychiatrist (TB). See Huddy et al. (2007) also.

### 2.3 Data collection

Information was obtained, with informed consent, from participants’ clinical records and clinical interview, as well as interviews with participants’ carers and relatives, where possible. Data gathered at the time of first presentation to services included basic demographic information, as well as performance on an array of clinical, cognitive and neuropsychological assessments. All researchers involved in data collection (the two research nurses mentioned above and a graduate research psychologist) received training to a high standard in the application of these measures. Data on ethnicity were not gathered routinely.

### 2.4 Measures

Psychotic symptoms were assessed using the SAPS and SANS (Andreasen, 1990), which were administered with an inter-rater reliability of ≥ 0.77 by IH and SM. The SAPS is a 34-item clinician-administered questionnaire, which divides symptoms into four sub-scales (hallucinations, delusions, bizarre behaviour and formal thought disorder), each of which is also given a global symptom severity score by the rater (global ratings). It is therefore comprised of 30 individual symptom ratings and four global ratings. The SANS is a 24-item clinician-administered questionnaire, which divides symptoms into five sub-scales (affective flattening or blunting, alogia, avolition-apathy, anhedonia-asociality, attention), also given global ratings. It is therefore comprised of 19 individual symptom ratings and five global ratings. The measures have been validated in recent-onset psychosis (Fulford et al., 2014) and correlate well with other symptom measures, e.g. the PANSS (*r* = 0.71–0.84) (van Erp et al., 2014).

### 2.5 Analyses

All analyses were undertaken using SPSS (version 22; SPSS Inc., Chicago, IL). In order to determine data factorability, data were assessed for sufficient correlation between items, excessively large inter-item correlations (*r* > 0.9), sphericity (Bartlett's test) and sampling adequacy (Kaiser, 1974)/anti-image correlation matrix diagonals > 0.5. Any failure to meet these checks are reported in the text.

In a global ratings level analysis the nine global ratings of the SAPS and SANS were exposed to a PCA following the methods described by John et al. (2003). Principal components were extracted if they had an eigenvector value > 1 using a VARIMAX rotation, leading to the extraction of orthogonal components.

In a symptom level analysis individual symptom item scores were exposed to a two-step analytic approach following the methods of Peralta and Cuesta (1999). The first-order PCA was first undertaken on all 49 individual symptom SAPS and SANS scores, followed by a second-order PCA on the principal component scores extracted from this first-order analysis.

For the first-order analysis, principal components were extracted if they had an eigenvector value > 1 using the OBLIMIN oblique rotation, since correlations were expected between symptom dimensions at this stage of analysis (Peralta et al., 1997). The second-order PCA was undertaken on principal component scores extracted from the first-order analysis using a VARIMAX rotation.

For all analyses undertaken individual items were retained/deemed to belong to an extracted dimension if they exhibited a loading of 0.4 or greater.

In order to assess the dependence of any findings on analytic approach all analyses were re-run using alternative methods of data reduction (whilst retaining dimensions using the Kaiser criterion method). Choices as to which methodological variants to include were made on the basis of the most common analyses adopted in the existing literature. This was because our intention was to explore the possible impact of these on reported findings rather than to undertake an exhaustive review of all possible statistical approaches. Thus, in addition to using the Kaiser criterion to define the number of factors to extract the scree method was also assessed. The effects of running EFA as an alternative to PCA was also explored. Four different estimation methods were used with the EFA: principle axis factoring, unweighted least squares, generalised least squares and maximum likelihood.

Finally, since a number of researchers have argued that the ‘Attention’ subscale should be excluded *a priori* from the SANS on the basis that attention is a neurocognitive domain, e.g. Blanchard and Cohen (2006), the primary PCAs were re-run without these subscale items. Since these analyses did not generate substantially different findings these data are presented in Supplementary Tables and discussed in brief only.

## 3 Results

### 3.1 Missing data and sample characteristics

Information as to the number of potential participants that were evaluated, screened and excluded was not routinely recorded throughout the study; consequently, these data are not available. Ultimately however, 345 participants met criteria for inclusion. Of these 345 full symptom data-sets (complete SAPS/SANS scores) were available for 335 cases; this formed the basis of all PCAs and EFAs reported (complete case analyses). Several additional variables are also reported for the patient sample, e.g. Age of Onset and Duration of Untreated Psychosis (Table 1). Whilst a number of individual cases were missing for these additional variables, with the exception of IQ (see below), this loss represented a small proportion of the total number of cases (< 3%).

**Table 1:** Demographic and clinical characteristics of the study sample. Statistics provided include the number of cases (N), the median and the inter-quartile range (IQR). Data are provided for the complete-case analysis data-set, i.e. participants for whom full symptom data were available (*N* = 335). Missing data (*n*/%) indicate the number and percentage of cases missing relative to this data-set. DUP = duration of untreated psychosis; NS-SEC = National Statistics Socio-Economic Classification system; SAPS = Scales for the Assessment of Positive symptoms; SANS = Scales for the Assessment of Negative symptoms; NART = National Adult Reading Test.

Variable	Level	*N*	Missing (*n*/%)	Median	IQR
Age at Assessment (years)	–	334	1 (0.29%)	24.07	20–30.13
Age at Onset (years)	–	326	9 (2.69%)	23	19–28
DUP (weeks)	–	330	5 (1.49%)	12	4–45
Gender	*All*	333	2 (0.6%)	–	–
	*Male*	218	–	–	–
	*Female*	115	–	–	–
NS-SEC	*All*	332	3 (0.9%)	–	–
	*Managerial and professional*	18	–	–	–
	*Intermediate occupations*	22	–	–	–
	*Routine and manual*	51	–	–	–
	*Student*	52	–	–	–
	*Unemployed*	189	–	–	–
Diagnosis	*All*	329	6 (1.79%)	–	–
	*Affective*	74	–	–	–
	*Non-affective*	255	–	–	–
SAPS total	–	335	0 (0%)	32	23–45
SANS total	–	335	0 (0%)	18	7–34
NART	–	267	68 (20.3%)	97	87–107
					

Participant characteristics are presented in Table 1, including the duration of untreated psychosis (DUP), calculated using the Nottingham Onset Scale (NOS) (Singh et al., 2005), socioeconomic status, defined on the basis of participant occupation using the National Statistics Socio-Economic Classification system (NS-SEC) (Rose and Pevalin, 2005) and premorbid IQ, assessed using the National Adult Reading Test (NART) (Lezak, 2004, Nelson and Wilson, 1991). The median age at assessment was 24.07 years with an inter-quartile range (IQR) of 20–30.13, the median age at onset was 23 years (IQR = 19–28), and the median DUP was 12 weeks (IQR = 4–45). With respect to socioeconomic status the vast majority of participants were unemployed (189 of 332 participants for whom these data were available). The majority of participants were recorded as having a diagnosis of non-affective psychosis (255 of 329 participants for whom these data were available) as opposed to an affective psychosis (*n* = 74). With respect to symptom severity, the median SAPS total score was 32 (IQR = 23–45) and the median SANS total score was 18 (IQR = 7–34). Finally, the median IQ score was 97 (IQR = 87–107), although these data were only available for a small section of the sample (*n* = 267); nonetheless, these data are presented to facilitate comparison with previous published studies.

### 3.2 Global ratings level analysis

**Fig. 1:**
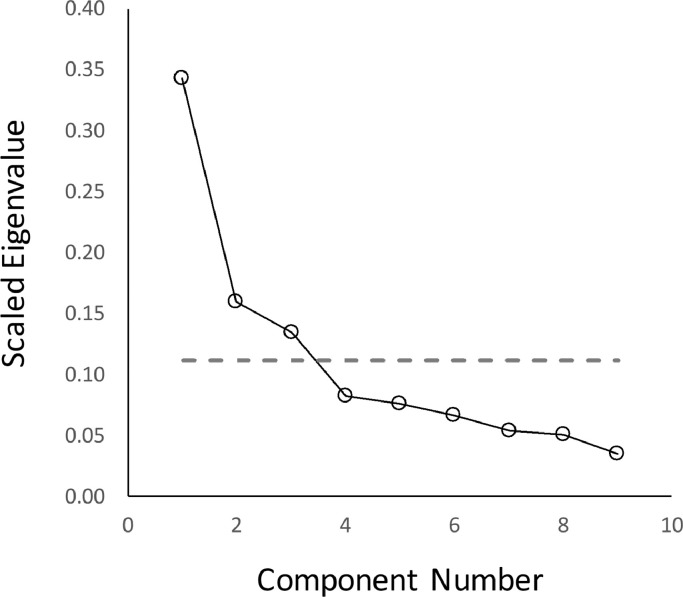
Scree plot for components identified in the global ratings level principal component analysis. Component numbers are plotted against the scaled eigenvalues for each eigenvector (eigenvalue divided by the total number of components). The dotted line represents the eigenvector > 1 line (i.e. 1 divided by the total number of components).

PCA of SAPS and SANS global ratings resulted in the extraction of three components with eigenvectors > 1; these explained 63.7% of the variance. Examination of the associated scree plot (see Fig. 1) indicated that if the scree test were used to determine the number of factors (instead of the Kaiser criterion method) the findings would be identical.

Extracted components mapped on to the classic triad of symptoms. Table 2 shows the component loadings. (See supplementary Table 1 also for the component score coefficient matrix). The first component (negative symptoms), which explained 34.3% of the variance in the data was comprised of loadings from affective flattening, alogia, avolition/apathy, anhedonia/asociality and attention. The second component (disorganisation symptoms), which explained 16% of the variance in the data, was comprised of loadings from delusions, bizarre behaviour and positive formal thought disorder. Finally, the third component (positive symptoms), which explained 13.5% of the variance in the data, was comprised of loadings from hallucinations and delusions.

**Table 2:** Component/factor loadings (rotated matrix) for all nine variables included in the global ratings level analyses. Solutions are shown for principal component analysis and exploratory factor analyses using principal axis factoring, unweighted least squares, generalised least squares and maximum likelihood extraction methods. Loadings > 0.4 in magnitude are shown in bold.

	Principal component analysis			Principal axis factoring			Unweighted least squares			Generalised least squares			Maximum likelihood		
	NEG	DIS	POS	NEG	DIS	POS	NEG	DIS	POS	NEG	DIS	POS	NEG	DIS	POS
*% variance explained*	34.3	16	13.5	34.3	16	13.5	34.3	16	13.5	34.3	16	13.5	34.3	16	13.5
(1) Hallucinations	0.12	−0.19	**0.82**	0.09	−0.08	**0.43**	0.09	−0.08	**0.43**	0.09	−0.09	**0.40**	0.08	−0.06	0.39
(2) Delusions	−0.06	**0.41**	**0.71**	−0.04	0.31	**0.61**	−0.04	0.31	**0.62**	−0.03	0.27	**0.55**	−0.05	0.30	**0.64**
(3) Bizarre behaviour	0.02	**0.82**	0.14	0.05	**0.63**	0.14	0.05	**0.63**	0.15	0.04	**0.62**	0.26	0.06	**0.55**	0.19
(4) Positive formal thought disorder	0.26	**0.74**	−0.11	0.24	**0.62**	−0.08	0.24	**0.63**	−0.08	0.22	**0.65**	−0.06	0.22	**0.70**	−0.09
(5) Affective flattening	**0.80**	0.01	0.11	**0.74**	0.03	0.13	**0.75**	0.03	0.13	**0.80**	−0.01	0.18	**0.79**	0.00	0.15
(6) Alogia	**0.78**	0.17	−0.04	**0.73**	0.17	−0.01	**0.73**	0.17	0.00	**0.76**	0.16	0.03	**0.76**	0.16	0.02
(7) Avolition / apathy	**0.68**	0.27	0.16	**0.61**	0.26	0.16	**0.61**	0.26	0.16	**0.58**	0.29	0.14	**0.58**	0.27	0.14
(8) Anhedonia / asociality	**0.74**	−0.14	0.05	**0.63**	−0.05	0.04	**0.63**	−0.05	0.04	**0.61**	0.00	−0.05	**0.58**	0.02	0.01
(9) Attention	**0.69**	0.29	−0.17	**0.62**	0.30	−0.14	**0.62**	0.30	−0.14	**0.59**	0.36	−0.20	**0.58**	0.34	−0.15

Highly similar results were obtained using EFA instead of PCA, irrespective of which extraction method was used, i.e. principle axis factoring, unweighted least squares, generalised least squares or maximum likelihood (see Table 2). The primary difference was that loadings were consistently lower for the EFAs than for PCA, a consequence of which is that several items just failed to reach the threshold for inclusion on some components, e.g. delusions on the disorganisation symptoms component (seen across all EFAs), and hallucinations on the positive symptoms component (seen in the maximum likelihood method only).

### 3.3 First-order symptom level analysis

Next, a PCA was undertaken on SAPS and SANS individual item scores. Three of the values on the diagonals of the anti-image correlation matrix were < 0.5, indicating items that did not share sufficient variance with other items to warrant inclusion. Consequently, these three items (persecutory delusions, delusions of sin or guilt and somatic delusions) were excluded and the analysis was rerun without them, i.e. using 46 of the full 49 individual symptom ratings.

**Fig. 2:**
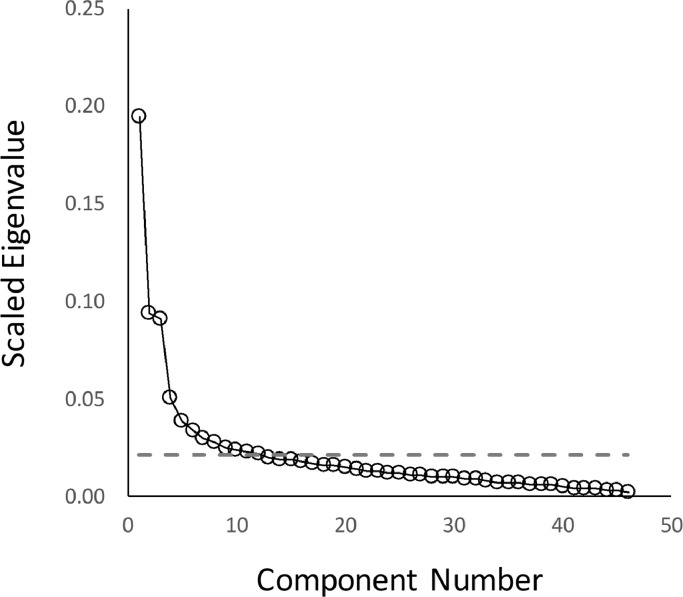
Scree plot for components identified in the first-order individual symptom level principal component analysis. Component numbers are plotted against the scaled eigenvalues for each eigenvector (eigenvalue divided by the total number of components). The dotted line represents the eigenvector > 1 line (i.e. 1 divided by the total number of components).

The first-order symptom level PCA resulted in the extraction of 11 components with eigenvectors > 1, which together explained 63.2% of the variance in the data. In contrast, it was not clear how many components should be extracted on the basis of the scree test since there was no clearly defined inflection point (see Fig. 2), and arguably, two inflection points: one that would lead to the extraction of ∼four or five components, and one that would lead to ∼11 or 12. Thus, the two methods of component retention produce highly divergent results in this example, since retention of the first four or five components would exclude most of the positive symptoms of psychosis (most of the hallucinations and delusions for example).

Table 3 shows the associated PCA structure with variable loadings. The 11 components extracted were named: (i) negative symptoms, (ii) thought disorder, (iii) delusions, (iv) social dysfunction, (v) bizarre behaviour, (vi) auditory hallucinations, (vii) grandiose and religious delusions, (viii) other hallucinations (ix) delusions of jealousy (comprised of a single item only), (x) alogia and inattentiveness, and (xi) other bizarre behaviour.

**Table 3:** Component loadings (structure) matrix for all 46 items included in the first-order principal component analysis. Loadings > 0.4 in magnitude are shown in bold. Note: three items of the original 49 were excluded as they did not meet assumptions of the analysis.

			F1	F2	F3	F4	F5	F6	F7	F8	F9	F10	F11
*% variance explained*			19.4	9.4	9.1	5	3.8	3.4	3	2.8	2.5	2.4	2.3
Hallucinations	SAPS-H1	Auditory	0.03	−0.08	0.29	−0.10	−0.09	**−0.81**	0.04	0.23	−0.09	0.04	0.10
	SAPS-H2	Voices commenting	0.07	−0.01	0.29	−0.15	−0.04	**−0.84**	0.01	0.23	0.08	0.01	−0.03
	SAPS-H3	Voices conversing	0.08	−0.03	0.27	−0.09	−0.09	**−0.82**	−0.09	0.14	0.06	0.05	−0.02
	SAPS-H4	Somatic or tactile	−0.12	−0.02	0.17	−0.03	−0.36	−0.38	−0.25	**0.45**	0.11	0.07	−0.08
	SAPS-H5	Olfactory	−0.02	0.02	0.30	−0.06	0.03	−0.14	−0.03	**0.72**	0.11	0.01	0.00
	SAPS-H6	Visual	0.01	0.03	0.14	−0.01	−0.08	−0.28	−0.04	**0.75**	0.03	−0.02	0.00
Delusions	SAPS-D2	Jealous	−0.02	−0.05	0.14	0.01	0.06	−0.04	0.03	0.12	**0.77**	−0.01	−0.06
	SAPS-D4	Grandiose	−0.15	0.21	0.10	0.12	0.30	0.01	**−0.69**	0.01	0.07	−0.06	−0.08
	SAPS-D5	Religious	−0.07	0.03	0.14	0.09	−0.09	−0.01	**−0.79**	0.05	−0.09	−0.04	−0.03
	SAPS-D7	Of reference	0.00	0.02	**0.45**	−0.14	−0.10	−0.25	−0.25	−0.05	0.33	0.13	0.34
	SAPS-D8	Of being controlled	0.04	0.07	**0.65**	−0.11	−0.25	−0.22	−0.32	0.21	0.05	0.15	0.03
	SAPS-D9	Of mind reading	0.05	0.11	**0.69**	−0.05	−0.11	−0.17	−0.13	0.10	0.25	0.11	0.08
	SAPS-D10	Thought broadcasting	0.05	0.07	**0.72**	−0.05	0.03	**−0.43**	−0.09	0.22	0.13	0.19	−0.01
	SAPS-D11	Thought insertion	0.10	−0.04	**0.72**	−0.03	−0.07	−0.32	−0.09	0.20	0.00	0.05	−0.10
	SAPS-D12	Thought withdrawal	0.13	0.12	**0.76**	−0.08	−0.01	−0.20	−0.01	0.28	0.01	−0.03	−0.06
Bizarre behaviour	SAPS-B1	Appearance	−0.09	0.11	−0.14	0.05	0.33	−0.09	−0.34	−0.07	0.05	−0.06	**−0.59**
	SAPS-B2	Social/sexual	0.05	0.19	0.00	0.03	**0.51**	0.10	**−0.42**	0.16	0.03	0.08	−0.18
	SAPS-B3	Aggressive/agitated	−0.02	0.01	−0.10	0.07	**0.77**	0.06	−0.01	−0.08	0.02	0.03	−0.08
	SAPS-B4	Repetitive/stereotyped	0.16	0.13	0.30	−0.05	−0.19	0.14	0.00	0.25	0.07	0.19	**−0.52**
Formal thought disorder	SAPS-P1	Derailment	0.12	**0.85**	0.10	−0.15	0.01	0.04	−0.07	0.02	−0.02	0.29	−0.13
	SAPS-P2	Tangentiality	0.15	**0.83**	0.05	−0.24	−0.02	0.01	−0.05	−0.05	0.06	0.31	−0.16
	SAPS-P3	Incoherence	0.13	**0.46**	0.15	−0.13	0.10	−0.05	0.01	−0.09	0.02	0.15	**−0.57**
	SAPS-P4	Illogicality	0.19	**0.79**	0.18	−0.24	0.10	0.05	−0.04	0.10	−0.05	0.24	−0.19
	SAPS-P5	Circumstantiality	0.17	**0.77**	0.15	−0.21	−0.03	0.00	−0.18	0.03	−0.02	0.16	−0.09
	SAPS-P6	Pressure of Speech	−0.19	**0.59**	−0.20	0.04	0.25	0.20	−0.21	−0.02	−0.08	−0.20	−0.21
	SAPS-P7	Distractible Speech	0.03	**0.61**	0.06	−0.03	0.35	0.04	−0.06	0.08	−0.24	0.24	−0.08
	SAPS-P8	Clanging	0.10	**0.61**	0.16	−0.15	0.19	0.07	−0.02	0.30	−0.31	0.00	−0.12
Affective flattening/blunting	SANS-1	Facial expression	**0.88**	0.09	0.08	−0.37	−0.06	−0.02	0.14	−0.05	−0.09	0.32	−0.03
	SANS-2	Spontaneous movements	**0.86**	0.12	0.13	−0.27	−0.07	−0.07	0.05	−0.02	−0.13	0.19	−0.09
	SANS-3	Expressive gestures	**0.93**	0.12	0.06	−0.36	−0.03	0.00	0.09	−0.01	−0.10	0.28	−0.07
	SANS-4	Eye contact	**0.56**	0.04	0.01	−0.24	0.09	−0.06	0.08	0.01	−0.08	0.37	−0.29
	SANS-5	Non-responsiveness	**0.87**	0.17	0.13	−0.35	0.07	−0.02	0.13	0.07	−0.02	0.29	−0.11
	SANS-7	Vocal Inflections	**0.87**	0.05	0.15	−0.36	0.02	−0.08	0.12	0.02	−0.02	0.30	−0.10
Alogia	SANS-9	Poverty of speech	**0.72**	−0.07	0.00	−0.19	0.05	−0.11	0.22	0.03	−0.15	**0.57**	0.00
	SANS-10	Poverty of speech content	0.36	**0.52**	0.01	−0.23	−0.05	0.07	0.00	0.05	−0.09	**0.53**	−0.12
	SANS-11	Blocking	**0.55**	0.26	0.26	0.03	0.07	0.02	0.15	0.15	−0.12	**0.64**	−0.01
	SANS-12	Latency of response	**0.68**	0.18	0.12	−0.04	0.05	−0.08	0.10	0.06	−0.19	**0.61**	0.04
Avolition/apathy	SANS-14	Grooming/hygiene	0.34	0.13	0.00	**−0.53**	0.26	−0.05	−0.07	−0.09	−0.36	0.27	**−0.43**
	SANS-15	Impersistence	0.23	0.24	0.11	**−0.68**	0.23	−0.09	−0.11	−0.02	−0.17	0.25	0.04
	SANS-16	Physical anergia	**0.52**	0.14	0.16	**−0.63**	−0.06	−0.15	0.02	0.00	−0.34	0.27	−0.06
Anhedonia/asociality	SANS-18	Recreational interest/ activity	0.35	0.10	0.08	**−0.70**	−0.19	−0.20	0.21	−0.03	−0.19	0.18	−0.01
	SANS-19	Sexual interest/activity	0.25	0.08	0.03	**−0.64**	−0.25	−0.15	0.09	0.15	0.00	−0.04	0.04
	SANS-20	Ability to feel intimacy	0.31	0.18	0.12	**−0.75**	−0.06	−0.04	0.19	0.04	0.21	0.20	−0.17
	SANS-21	Relationships	**0.45**	0.24	0.06	**−0.81**	−0.02	−0.05	0.16	0.07	0.14	0.32	−0.08
Attention	SANS-23	Social inattentiveness	0.29	0.35	0.13	**−0.45**	0.10	0.00	0.02	−0.04	−0.02	**0.73**	−0.16
	SANS-24	Inattentiveness during testing	0.39	**0.44**	0.04	−0.39	0.10	0.02	0.03	−0.02	−0.09	**0.66**	−0.29

Additional analyses were undertaken to determine the effects of using EFA as opposed to PCA (Supplementary Tables 2–5). Since the models would not converge (within 9999 iterations) using oblique rotation methods (OBLIMIN or PROMAX), an orthogonal rotation (VARIMAX) was used instead. This was not planned for *a priori*, and it is not clear why convergence did not occur: communalities were all < 1, ruling out the possibility of a Heywood Case, and all tests of data factorability were passed.

The main findings were highly robust, particularly for the lower numbered components with multiple loadings, which showed high consistency across EFA methods and only minor differences in their ordering. For example, negative symptoms, thought disorder, social dysfunction and delusions consistently emerged as the first four components, with negative symptoms consistently the first to be extracted, thought disorder consistently the second, and social dysfunction and delusions variably extracted third and fourth, or fourth and third, respectively. Other minor differences were due to individual items failing to cross the > 0.4 inclusion threshold, as well as the relative instability of components with few item loadings.

Finally, see Supplementary Table 6 for details on the effects of re-running the first-order PCA without items relating to the Attention subscale. Eleven components were again extracted, which together explained 63.9% of the variance. These were sufficiently similar in their pattern of item loadings that the first nine components extracted were labelled identically to the basic PCA (see Supplementary Table 7 also). Components ten and 11 also showed similarities across analyses, but were reversed in their relative ordering. Fundamentally therefore, removal of items relating to attention had very little impact on the pattern of findings.

### 3.4 Second-order symptom level analysis

**Fig. 3:**
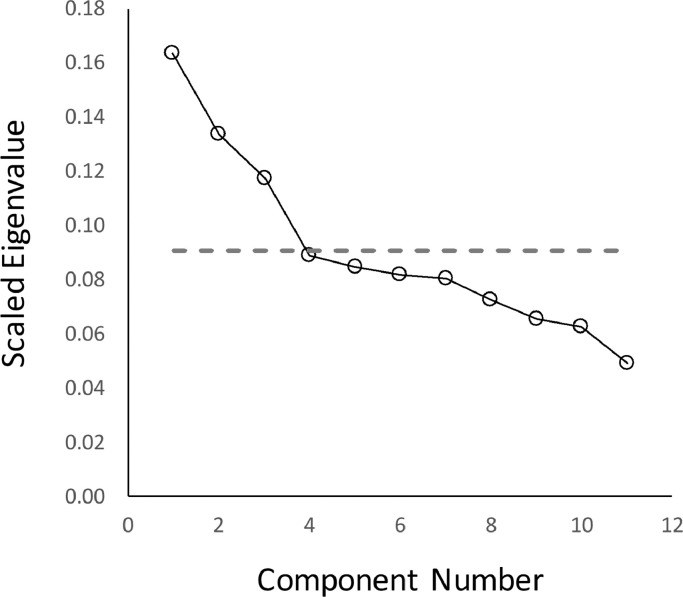
Scree plot for components identified in the second-order symptom level principal component analysis. Component numbers are plotted against the scaled eigenvalues for each eigenvector (eigenvalue divided by the total number of components). The dotted line represents the eigenvector > 1 line (i.e. 1 divided by the total number of components).

Next a second-order PCA was undertaken on the component scores to have emerged from the first-order symptom level analysis. Three components were extracted with eigenvectors > 1, explaining 41.5% of the variance. Examination of the associated scree plot (Fig. 3), indicated that use of the scree test resulted in identical findings.

Table 4 (columns 1–4) shows the associated PCA structure with variable loadings. The first component (negative symptoms), which explained 16.4% of the variance, showed loadings from the negative symptoms, social dysfunction and alogia and inattentiveness components derived from the first-order PCA. The second component (positive symptoms), which explained 13.3% of the variance, showed loadings from delusions, auditory hallucinations and other hallucinations. The third component (disorganisation symptoms), which explained 11.7% of the variance, showed loadings from thought disorder, bizarre behaviour, grandiose and religious delusions and other bizarre behaviours.

**Table 4:** Component / factor loadings (rotated matrix) for all 11 variables included in the second-order analyses. The coefficients of components 4, 6, 7 and 11 have been inverted to aid interpretation. This is appropriate, since in the first-order PCA the loadings associated with these four components were negative, i.e. of a different sign to the other components. Loadings > 0.4 in magnitude are shown in bold.

	Principal Component Analysis			Principal Axis Factoring			Unweighted Least Squares			Generalised Least Squares			Maximum Likelihood		
	NEG	POS	DIS	NEG	POS	DIS	NEG	POS	DIS	NEG	DIS	POS	NEG	POS	DIS
*% variance explained*	16.4	13.3	11.7	16.4	13.3	11.7	16.4	13.3	11.7	16.4	13.3	11.7	16.4	13.3	11.7
(1) Negative Symptoms	**0.78**	−0.02	−0.01	**0.78**	−0.01	−0.03	**0.78**	−0.01	−0.03	**0.87**	−0.05	−0.01	**0.87**	−0.02	−0.05
(2) Thought Disorder	0.29	0.04	**0.62**	0.20	0.05	**0.54**	0.20	0.05	**0.54**	0.17	**0.81**	0.02	0.16	0.04	**0.74**
(3) Delusions	0.16	**0.72**	0.05	0.12	**0.63**	0.05	0.12	**0.63**	0.05	0.12	0.07	**0.62**	0.13	**0.63**	0.06
(4) Social dysfunction	**0.63**	0.10	0.00	0.40	0.09	0.06	0.40	0.09	0.06	0.37	0.11	0.09	0.37	0.08	0.11
(5) Bizarre behaviour	−0.08	−0.25	**0.57**	−0.03	−0.16	0.29	−0.03	−0.16	0.29	0.00	0.16	−0.17	0.00	−0.16	0.19
(6) Auditory hallucinations	0.09	**0.62**	−0.17	0.06	**0.41**	−0.12	0.06	**0.41**	−0.12	0.04	−0.10	**0.43**	0.05	**0.41**	−0.12
(7) Grandiose and religious delusions	−0.34	0.32	**0.51**	−0.18	0.17	0.25	−0.18	0.17	0.25	−0.15	0.16	0.15	−0.15	0.15	0.17
(8) Other hallucinations	0.00	**0.57**	0.09	0.02	0.33	0.04	0.02	0.33	0.04	0.02	0.05	0.33	0.02	0.33	0.04
(9) Jealous delusions	−0.22	0.29	−0.11	−0.12	0.15	−0.07	−0.12	0.15	−0.07	−0.13	−0.06	0.16	−0.13	0.16	−0.06
(10) Alogia and inattentiveness	**0.64**	0.06	0.17	**0.45**	0.07	0.16	**0.45**	0.07	0.16	**0.42**	0.15	0.08	**0.42**	0.08	0.16
(11) Other bizarre behaviour	0.12	−0.02	**0.55**	0.10	−0.02	0.28	0.10	−0.02	0.28	0.10	0.19	−0.03	0.10	−0.03	0.21

Use of EFA instead of PCA (Table 4 columns 5–16) resulted in three highly similar factors that could also be labelled negative, positive and disorganisation symptoms. The main differences between the PCA and EFAs was that in the latter, the positive symptoms factor did not include a loading from other hallucinations and the negative symptoms factor did not include a loading from social dysfunction; this was consistent across factor analytic methods used. Further, in the EFAs the disorganisation symptom factor was uniquely associated with symptoms of thought disorder, i.e. there were no significant loadings from bizarre behaviour and grandiose/religious delusions.

Finally, see Supplementary Table 7 for details on the effects of re-running the second-order PCA using the output of the first-order analysis which did not include items relating to the Attention subscale. Once again, the findings were very similar to those reported when items relating to attention were retained. Three components were again extracted, which together explained 40.9% of the variance. These were labelled negative, positive and disorganisation components, and exhibited a near identical pattern of item loadings to the basic PCA.

## 4 Discussion

Consistent with hypothesis one (dependence on level of analysis), the data suggest that whilst symptom level analysis of the SAPS and SANS results in ten + inter-correlated first-order components, global ratings level analysis results in a three component solution (Toomey et al., 1997). The findings also support the robustness of the triadic syndrome model (Grube et al., 1998, Smith et al., 1998). Thus, positive, negative and disorganisation symptom dimensions emerged from analyses of SAPS and SANS global ratings as well as second-order analyses of individual item ratings, irrespective of data reduction and component retention method.

Consistent with hypothesis two (hierarchical symptom structure) the findings also support a complex hierarchical structure to the symptom data, with the aforementioned 10 + first-order factors being subsumed by three-to-four second-order factors (Peralta and Cuesta, 1999). Infact, the findings are remarkably similar to those reported by Peralta and Cuesta (1999); this, despite a considerable difference in population sample (first-episode versus multi-episode psychosis), a fact that points to the robustness of the findings/symptom structure revealed. Thus, whilst Peralta and Cuesta (1999) reported 12 first-order components, which explained 66% of the variance, we report 11 first-order components, which explain 63%, the first four of which were nearly identical across studies. The two sets of studies also rendered highly similar, second-order components, with Peralta and Cuesta (1999) reporting four second-order dimensions, which explained 54% of the variance, where we report three, which together explained 41%. Whilst the first three components mapped on to the classic triad of syndromes (positive, negative and disorganisation), the fourth component identified by Peralta and Cuesta (1999) was comprised of a single loading only (‘other delusions’).

Whilst a minority of the first-order components identified could be mapped almost directly onto SAPS and SANS global rating sub-scales, e.g. component two (thought disorder), other components identified either split or cut across categories with loadings from across multiple sub-scales, e.g. component six (auditory hallucinations), which included items from the hallucinations sub-scale (including auditory hallucinations) as well as the delusions subscale (thought broadcasting). This lack of direct correspondence is perhaps not surprising given that the nine global rating sub-scales of the SAPS/SANS were in fact generated on the basis of clinical opinion and subjective experience (Andreasen, 1990, Andreasen and Olsen, 1982). This questions the validity of the SAPS and SANS sub-scales and has led some to call for their re-structuring along more empirically-defined lines (de Leon et al., 1993, Keefe et al., 1992, Vazquez-Barquero et al., 1996).

### 4.1 Dependence on component number estimation method

Whilst the same core symptom structure was revealed across analyses the choice of component retention method did have some impact on findings (scree test versus Kaiser criterion method). Whilst the two techniques rendered identical results for the global ratings and second-order symptom level analyses, findings for the first-order symptom level analysis were less stable. The scree test did not yield a definitive number of components: inflection points were multiple and ambiguous; see Ledesma (2007) and Zwick and Velicer (1986) for discussion. One possible inflection point would have yielded a comparable number of components to the Kaiser criterion method (∼11 or 12), whilst the other would have yielded far fewer (∼four or five). The choice of retention method thus has profound implications on the findings since the exclusive retention of the first four components would result in the loss of all information relating to hallucinations as well as a subset of delusions (see Table 3). This would seem to represent an unjustifiable loss of information from a clinical perspective. In contrast, retention of the full 11–12 components (e.g. as indicated by the Kaiser criterion method) would result in the inclusion of components with few item loadings (sometimes only one, e.g. Table 3 component 9), or components that combine seemingly unrelated symptoms that make little sense clinically (e.g. Table 3, component 7).

The findings therefore support the notion that discrepancies in the literature as to the precise number of psychotic components may be driven, in part, by differences in the methods used to determine the number of factors. Further, they are consistent with Peralta and Cuesta's (2001) review of the literature, which found that the use of the scree method consistently resulted in the extraction of around half the number of dimensions rendered by the Kaiser criterion method.

### 4.2 Dependence on data reduction method

The choice of data reduction method did not impact drastically upon the findings. PCA and EFA generated near-identical results in the global ratings and symptom level analyses. There was, however, a consistent trend for lower loadings using EFA relative to PCA, with several items failing to reach the threshold for inclusion as a result.

Peralta and Cuesta similarly concluded that the core structure underlying psychotic symptoms (as revealed by common symptom measures) is relatively robust to changes in data reduction method. They found near-identical effects of using EFA (with principal axis factoring) or PCA to analyse SAPS and SANS data in a sample of 660 patients with psychosis (Peralta and Cuesta, 1999), and further, in a review of the literature noted robust factor solutions across a range of studies using different data reduction methods (Peralta and Cuesta, 2001).

### 4.3 Model utility and validity

The data reported strongly suggest that a simple dichotomy between positive and negative symptoms, which underlies the construction of the SAPS and SANS, does a poor job of capturing the full complexity of the underlying symptom structure. Thus, at the very least, it is clear that the symptoms of disorganisation cannot be lumped together on either positive or negative dimensions, but instead, represent a distinct syndrome or cluster; see discussion in Dazzi et al. (2016) for example. Beyond this, however, it is not clear which level of description described here offers the most useful account of psychotic symptom structure: the first-order model, which describes 10 + dimensions, retaining much of the complexity of the original data-set, or a higher-order (more parsimonious) model based on the classic triad of positive, negative and disorganisation symptoms. To address this question as it related to their own data, Peralta and Cuesta (1999) turned to the amount of variance explained by each level of the model (first-order and second-order). Thus, whilst the data reported here show that the 11 first-order components accounted for 63.1% of the variance in the 46 symptoms included in the model, the three second-order components accounted for 41.5% of the first-order components. This implies that the second-order components only accounted for 26.2% of the variance in individual SAPS/SANS scores (41.5% of 63.2%). This represents a considerable loss of variance, and suggests that the positive, negative and disorganisation symptom clusters, by themselves, do a relatively poor job of capturing the full richness of psychotic symptoms.

This trade-off between parsimony and completeness of description is inevitable, however, and the relative utility of each model will depend on the purpose/s for which they are being used. One might argue, therefore, that clinical/professional judgement should play a crucial role in deciding how many -and which- components should be retained, although this would inevitably introduce a further stage of subjectivity to the analyses. For example, a measure/model that is used in a clinical context, should be of clinical utility, able to assist in the processes of assessment, treatment and outcome monitoring. One might question, for example, to what extent component nine in the first-order item-level analysis (comprised of a single item: delusions of jealousy) adds anything of clinical utility to the model (Table 3). In contrast, models employed in research may require a distinct set of utilities, although there may be some overlap, particularly where research is applied and of direct clinical relevance.

With this in mind, the triadic symptom model is robust, highly reproducible (including across different measures) and, with only three scores to define it, easy to manage with good face validity (Grube et al., 1998, Smith et al., 1998). For research purposes, such as the investigation of associations between symptom dimensions and defined risk factors, clinical outcomes and treatment effects can be tested and interpreted without a large inflation in the risk of a type one error (Allardyce et al., 2007a, Oher et al., 2014, Wickham et al., 2014), as would be the case if a more complex multi-dimensional model were used. However, this loss of information inevitably risks missing patterns of association operating at a finer scale of analysis, e.g. between risk factors and individual symptoms. (Note: a similar discussion in the literature has arisen around the benefits versus costs and trade-offs involved in using longer versus shorter versions of common symptom measures such as the PANSS; see Lindenmayer (2017) and Lin et al. (2018) for example.)

If a system of symptom classification is to be truly valid, however, one might argue that its structure should reflect something meaningful about the aetiology, course or treatment-responsiveness of symptoms (for example), rather than mere statistical artefact. In support of the former, there is some evidence to suggest that positive, negative and disorganisation symptom clusters are predictive of differences in clinical course and outcome (Allardyce et al., 2007a, Austin et al., 2013). Further, they may be associated with distinct neuropsychological profiles (Aderibigbe and Gureje, 2008, Basso et al., 1998, Liddle and Morris, 1991) and partially separable patterns of structural and functional brain abnormalities (Kaplan et al., 1993, Koutsouleris et al., 2008, Mozley et al., 1994, Schröder et al., 1995, Zhang et al., 2015). However, components extracted at a finer grain of analysis, e.g. the delusions, bizarre behaviour and social dysfunction to emerge from the first-order symptom level analysis, may also show unique patterns of association with defined risk factors and treatment outcomes; see Bentall et al. (2012) for example. Further research is needed therefore, to determine which items and symptom clusters embedded in commonly used symptom measurement tools correlate/predict other parameters that are of genuine importance to our understanding of psychosis, and conversely, which items/clusters should be omitted from these measures.

In this regard it is worth noting that, to date, no formal assessment of the relative validity (e.g. discriminant validity or predictive validity) of higher-order versus lower-order factors has been undertaken. Irrespective, it would seem essential that this question be addressed if dimensional systems of classification are to be adopted more widely in clinical and research practice (Morris and Cuthbert, 2012, Parker, 2014). In seeking to validate different models, however, it would be a mistake to assume that all dimensions extracted necessarily reflect mechanisms that reside at a single, common level. Returning to the example above, whilst it might be possible to link delusions to defined neurobiological substrates, hypothetically, one might find that other symptoms, e.g. social dysfunction, show much less specificity in their association; instead they might relate to multiple factors and processes operating at distinct levels, e.g. dysfunction within defined cortical networks underpinning social cognition, but also, behavioural, inter-personal –and potentially even sociological- processes. Hence, in validating distinct models of symptoms it will be important to seek their correlates at multiple levels (from the neurophysiological to the social), whilst paying close attention to the meaning of extracted dimensions alongside their statistical properties.

### 4.4 Implications and future research

Within the fields of psychosis research (Morris and Cuthbert, 2012), classification (American Psychiatric Association 2013, Parker, 2014) and clinical practice (The British Psychological Society, 2014), it has been argued that the current system of nosology is far from optimal, and that a shift towards a dimensional model would be beneficial. However, it is arguable that such a shift would only be fruitful and meaningful to the extent that the dimensional system adopted is robust, e.g. reliable and valid across broad variations in age, gender, ethnicity, culture, diagnosis, stage of illness, duration without treatment and methods of administration; see Anderson et al., 2017, Rabinowitz et al., 2017 and Lehoux et al. (2009) for example.

Future studies are therefore needed to explore the extent to which symptom models vary (or remain consistent) across different population samples. The results reported here, however, suggest that differences in statistical methodology may also contribute to variations in findings across studies; see Peralta and Cuesta (2001) and Toomey et al. (1997). In order to distinguish between this kind of statistical artefact and informative ‘treatment effects’, a number of approaches can be employed. As a minimum, authors should make explicit the precise analytic methods they use, as well as the reasoning behind their choices; thus, even seemingly minor decisions such as the choice of data factorability tests employed are likely to impact upon the findings (see Section 3.3 for example). Ideally however, sensitivity analyses should be undertaken (as reported here), so that the effects of changing the methodology are tested within the same data-set.

Whilst we have explored the effects of varying data reduction, factor extraction and rotation methods in a simple (uni-dimensional) model as well as a two-tiered hierarchical model, other modelling approaches have been used and should be explored further. For example, Bentall and colleagues have shown that a non-hierarchical bifactor model, in which a single ‘general’ psychosis factor competes with five correlated symptom factors to describe variance in symptoms scores, provides a better fit to psychotic symptoms than uni-dimensional or two-tiered hierarchical models of the kind tested here and commonly found in the literature. Further, this held true for patients with diagnoses of affective and non-affective psychosis, as well as participants from the general population (Reininghaus et al., 2016, Reininghaus et al., 2013, Shevlin et al., 2017). To facilitate such comparisons and foster transparency journals should demand open-access to data; information can then be aggregated across studies, and discrepancies due to variations in approach tested (Gewin, 2016). It is worth noting also, that debate as to the relative merit of diagnostic versus dimensional systems of classification, as well as the validity of hierarchical versus non-hierarchical (e.g. bifactor) models of mental health difficulties, is not restricted to the study of psychosis, but is mirrored in more general models of psychopathology also; see Lahey et al. (2018) and Kotov et al. (2018) for discussion. Consequently, the findings reported here may have broader relevance outside the field of psychosis. To test this however, future studies that integrate data from across multiple diagnostic categories are needed in addition to in-depth meta-analyses/systematic reviews of the field; see Waszczuk et al. (2017) for example.

Finally, basic data reduction approaches of this kind should also be used in conjunction with a broad array of functional, clinical, cognitive, neuropsychological, psychosocial and environmental indices, in order to determine the neurobiological and etiological underpinnings (or otherwise) of derived models, as well as their clinical and prognostic utility. It is likely that multi-disciplinary research of this kind will deepen our understanding of how psychosis symptoms emerge and are maintained, and hence, lend itself to the development of novel treatments and interventions that target specific symptoms or symptoms clusters; see, for example, Pontillo et al., 2016, Freeman and Garety, 2006 and Remington et al. (2016).

### 4.5 Limitations

There are a number of potential limitations to this study. First, due to a lack of correlation with other variables a number of items were excluded from the first-order symptom level analyses. Whilst this approach was methodologically sound, one of the symptoms to be discarded was persecutory delusions, one of the most commonly reported in psychosis. It is not clear why this was the case; however, the exclusion of this symptom should be considered in future analyses of these data and/or use of the statistical models generated (see Supplementary Table 1). For example, it may limit the extent to which extracted components would be expected to correlate with other variables commonly associated with paranoia, e.g. measures of attributional biases (Bentall et al., 2001).

Second, the study only explored the structure of the SAPS and SANS, symptom measures that do not include items relating to mood disorder. As a result, the findings reported are limited in the extent to which they can be compared directly to other common psychosis symptom measures that include a more comprehensive list of items, e.g. the PANSS (Kay et al., 1987) and operational criteria checklist for psychotic illnesses (OCCPI: McGuffin et al., 1991). Thus, PCA/EFA of PANSS and OCCPI ratings typically render a five-factor solution that includes manic and depressive symptom dimensions (van der Gaag et al., 2006, Wallwork et al., 2012) in addition to the classic triad of symptoms (Peralta and Cuesta, 2001). Whilst this does not challenge the robustness of the positive, negative and disorganisation symptoms as a core underlying structure, it does suggest that the triadic syndrome model (underlying the SAPS/SANS for example) may not capture the full range of symptoms associated with psychotic illness. However, it is worth noting that despite differences in their design, symptom measures such as the PANSS and SAPS/SANS have still been found to converge; see van Erp et al. (2014) for example.

With respect to data gathering, one potential limitation that was raised by a reviewer is that participants recruited to the study were diagnosed by nurses rather than a psychiatrist or clinical psychologist. However, as mentioned in the Methods section, these were psychiatric research nurses who were extremely experienced in their field and trained to a high level in the administration of all relevant screening, diagnostic and symptom measurement tools. Further, and critically, they showed high inter-rater reliability on the SAPS and SANS.

Another limitation to the study is the possible confounding effects of medication. Thus, it is unclear whether the findings reported would be different in patients with no history of exposure to medication. Although individuals were excluded from the study if they had been taking antipsychotics for more than 12 weeks, a number of reviews have in fact shown effects on symptoms, e.g. insight (Mattila et al., 2017), after a considerably shorter period of medication use (Agid et al., 2006). However, it is worth noting that the core triadic syndrome structure has been demonstrated across a wide range of studies including patients with recent-onset as well as chronic psychotic illness, patient samples that are likely to have varied considerably with regards to medication history (Peralta and Cuesta, 2001). Further, follow-up/longitudinal studies suggest that whilst at the level of the individual symptom dimension scores may shift with time (i.e. symptom severity may shift) (Dragioti et al., 2017, Edgar et al., 2014), within a patient sample the core symptom structure remains relatively robust (Reichenberg et al., 2005), presumably despite an accumulating history of medication use. Nonetheless, we cannot rule out the possibility that the findings reported may have differed if characteristics of the population sample tested had differed considerably, e.g. with respect to medication history and/or DUP.

Finally, at a methodological level, it is worth noting that all dimension reduction approaches used here (i.e. variants of the EFA and PCA) are based on Pearson's correlation matrices. Whilst, to the authors’ knowledge this reflects the full range of approaches that have been used to analyse the structure of psychotic symptoms in the extant literature, Pearson's correlation matrices are in fact intended for use with interval or ratio data, which cannot be assumed with self-report Lickert scales such as the SAPS and SANS; nonetheless, this is common practice within the biological and social sciences (Gilley and Uhlig, 1993). Thus, whilst analyses included in this study were limited to basic EFA and PCA (in line with the stated aim of exploring how *commonly* reported statistical practices may have shaped discrepancies in the extant literature), future studies should examine the impact of using alternative data reduction approaches that do not rely on correlation matrices that assume normality. Thus, it is possible that the use of an alternative correlation matrix, e.g. one based on Spearman‘s rank correlation coefficients, would further change the pattern of findings to emerge.

## Appendix Supplementary materials

Image, application 1

Image, application 2
