# Supplementary material for: The component structure of the scales for the assessment of positive and negative symptoms in first-episode psychosis and its dependence on variations in analytic methods
Source: Psychiatry Res. 2018 Dec;270:869–79. doi: 10.1016/j.psychres.2018.10.046 (PMC6299359; doi:10.1016/j.psychres.2018.10.046)
Supplement: Supplementary file 2 [file mmc2.docx]

**Supplementary Table 1 Component Score Coefficient Matrix.** The component score coefficient matrix is shown for the global ratings level principal component analysis (PCA) undertaken. These data are included since they can be used in subsequent studies to generate negative, disorganisation and positive syndrome scores (i.e. component scores) from SAPS and SANS global ratings. According to our analyses / data-set, these subscale scores can then be used in place of all nine global ratings, whilst retaining 63.7% of the information. Thus, for each participant, a component score can be calculated for each of the components (NEG, DIS, POS) by multiplying (and subsequently summing) standardized (i.e. Z-transformed) global ratings values by the components’ score coefficients. For example, if a participant had (standardised) global ratings scores of -1.66, 0.88, 0.82, -0.93, -1.08, -1.02, -0.14, -0.04 and -0.65, according to the PCA undertaken, they would have a negative component score of -1.06, i.e. (-1.66*0.06) + (0.88*-0.09) + (0.82*-0.11) + (-0.93*0) + (-1.08*0.3) + (-1.02*0.28) + (-0.14*0.22) + (-0.04*0.3) + (-0.65*0.23).

|  | Principal Components  Analysis | | |
| --- | --- | --- | --- |
|  | NEG | DIS | POS |
| (1) Hallucinations | 0.06 | -0.20 | 0.66 |
| (2) Delusions | -0.09 | 0.23 | 0.54 |
| (3) Bizarre behaviour | -0.11 | 0.54 | 0.05 |
| (4) Positive formal thought disorder | 0.00 | 0.47 | -0.14 |
| (5) Affective flattening | 0.30 | -0.11 | 0.07 |
| (6) Alogia | 0.28 | 0.01 | -0.06 |
| (7) Avolition/apathy | 0.22 | 0.08 | 0.10 |
| (8) Anhedonia/asociality | 0.30 | -0.20 | 0.03 |
| (9) Attention | 0.23 | 0.11 | -0.17 |
|  |  |  |  |

**Supplementary Table 2 Component loadings (rotated) matrix for all 46 items included in the primary first-order exploratory factor analysis using the principal axis factoring extraction method.** Loadings >0.4 in magnitude are shown in full contrast. Note: three items of the original 49 were excluded as they did not meet assumptions of the analysis.

|  |  |  | F1 | F2 | F3 | F4 | F5 | F6 | F7 | F8 | F9 | F10 | F11 |
| --- | --- | --- | --- | --- | --- | --- | --- | --- | --- | --- | --- | --- | --- |
| *% variance explained* |  |  | 19.4 | 9.4 | 9.1 | 5 | 3.8 | 3.4 | 3 | 2.8 | 2.5 | 2.4 | 2.3 |
| Hallucinations | SAPS-H1 | Auditory | 0.01 | -0.08 | 0.09 | 0.23 | **0.71** | -0.07 | -0.04 | 0.15 | -0.02 | 0.09 | 0.06 |
|  | SAPS-H2 | Voices commenting | 0.05 | -0.02 | 0.12 | 0.23 | **0.79** | 0.04 | -0.04 | 0.15 | -0.05 | -0.01 | -0.09 |
|  | SAPS-H3 | Voices conversing | 0.06 | -0.03 | 0.04 | 0.24 | **0.68** | -0.03 | 0.05 | 0.08 | 0.04 | -0.06 | 0.02 |
|  | SAPS-H4 | Somatic or Tactile | -0.10 | 0.01 | 0.01 | 0.21 | 0.25 | -0.16 | 0.14 | 0.31 | 0.06 | -0.07 | 0.02 |
|  | SAPS-H5 | Olfactory | -0.03 | 0.02 | 0.04 | 0.26 | 0.10 | 0.03 | -0.01 | **0.45** | -0.02 | 0.02 | -0.07 |
|  | SAPS-H6 | Visual | 0.00 | 0.02 | 0.00 | 0.13 | 0.20 | -0.04 | 0.02 | **0.56** | -0.02 | 0.01 | -0.03 |
| Delusions | SAPS-D2 | Jealous | -0.03 | -0.05 | 0.01 | 0.16 | 0.01 | 0.05 | -0.04 | 0.06 | 0.00 | -0.02 | -0.34 |
|  | SAPS-D4 | Grandiose | -0.14 | 0.17 | -0.11 | 0.12 | -0.01 | 0.33 | **0.46** | -0.02 | -0.03 | -0.05 | -0.05 |
|  | SAPS-D5 | Religious | -0.06 | 0.02 | -0.07 | 0.15 | -0.01 | 0.06 | **0.70** | 0.05 | -0.02 | -0.01 | 0.08 |
|  | SAPS-D7 | Of reference | -0.02 | 0.00 | 0.10 | **0.40** | 0.16 | -0.11 | 0.15 | -0.09 | 0.01 | 0.05 | -0.18 |
|  | SAPS-D8 | Of being controlled | 0.01 | 0.07 | 0.07 | **0.59** | 0.09 | -0.13 | 0.22 | 0.13 | 0.05 | 0.00 | 0.05 |
|  | SAPS-D9 | Of mind reading | 0.02 | 0.09 | 0.01 | **0.60** | 0.07 | -0.06 | 0.06 | 0.03 | 0.01 | 0.03 | -0.17 |
|  | SAPS-D10 | Thought broadcasting | 0.04 | 0.06 | -0.01 | **0.66** | 0.26 | 0.06 | -0.03 | 0.12 | 0.11 | 0.00 | -0.06 |
|  | SAPS-D11 | Thought insertion | 0.08 | -0.04 | 0.00 | **0.63** | 0.19 | 0.02 | 0.01 | 0.11 | -0.01 | -0.01 | 0.05 |
|  | SAPS-D12 | Thought withdrawal | 0.08 | 0.09 | 0.04 | **0.65** | 0.08 | 0.02 | -0.05 | 0.18 | -0.11 | 0.02 | 0.02 |
| Bizarre behaviour | SAPS-B1 | Appearance | -0.06 | 0.12 | -0.07 | -0.08 | 0.05 | **0.42** | 0.14 | 0.03 | 0.11 | -0.25 | 0.05 |
|  | SAPS-B2 | Social/sexual | 0.05 | 0.17 | -0.05 | 0.02 | -0.07 | **0.42** | 0.20 | 0.06 | -0.01 | 0.02 | -0.02 |
|  | SAPS-B3 | Aggressive/agitated | 0.00 | 0.00 | -0.04 | -0.09 | -0.03 | **0.52** | -0.06 | -0.10 | -0.01 | 0.08 | -0.05 |
|  | SAPS-B4 | Repetitive/stereotyped | 0.14 | 0.15 | 0.05 | 0.21 | -0.09 | 0.03 | 0.02 | 0.17 | 0.06 | 0.01 | -0.02 |
| Pos formal thought disorder | SAPS-P1 | Derailment | 0.05 | **0.84** | 0.04 | 0.05 | -0.01 | -0.05 | 0.03 | -0.01 | 0.08 | 0.02 | -0.01 |
|  | SAPS-P2 | Tangentiality | 0.08 | **0.84** | 0.11 | 0.02 | 0.02 | -0.08 | 0.01 | -0.08 | 0.14 | -0.06 | -0.07 |
|  | SAPS-P3 | Incoherence | 0.10 | **0.43** | 0.06 | 0.09 | 0.01 | 0.18 | -0.04 | 0.02 | 0.15 | -0.17 | 0.04 |
|  | SAPS-P4 | Illogicality | 0.12 | **0.74** | 0.13 | 0.11 | -0.05 | 0.07 | -0.01 | 0.08 | 0.04 | 0.00 | 0.02 |
|  | SAPS-P5 | Circumstantiality | 0.10 | **0.72** | 0.10 | 0.10 | 0.01 | -0.04 | 0.13 | -0.01 | -0.02 | -0.06 | 0.00 |
|  | SAPS-P6 | Pressure of Speech | -0.20 | **0.52** | -0.04 | -0.20 | -0.11 | 0.25 | 0.12 | -0.01 | -0.19 | -0.07 | 0.01 |
|  | SAPS-P7 | Distractible Speech | -0.01 | **0.55** | 0.02 | 0.01 | 0.01 | 0.26 | -0.01 | -0.02 | -0.05 | 0.24 | 0.11 |
|  | SAPS-P8 | Clanging | 0.03 | **0.53** | 0.12 | 0.07 | -0.03 | 0.15 | 0.01 | 0.20 | -0.18 | 0.14 | 0.14 |
| Affective flattening / blunting | SANS-1 | Facial expression | **0.84** | 0.06 | 0.22 | 0.02 | -0.01 | -0.10 | -0.03 | -0.08 | 0.01 | -0.02 | 0.00 |
|  | SANS-2 | Spontaneous movements | **0.81** | 0.10 | 0.13 | 0.08 | 0.03 | -0.08 | 0.04 | -0.05 | -0.12 | -0.06 | 0.11 |
|  | SANS-3 | Expressive gestures | **0.91** | 0.09 | 0.20 | -0.01 | -0.03 | -0.06 | 0.03 | -0.02 | -0.08 | -0.03 | 0.01 |
|  | SANS-4 | Eye contact | **0.50** | 0.06 | 0.16 | 0.00 | 0.03 | 0.12 | -0.06 | 0.02 | 0.15 | 0.02 | 0.08 |
|  | SANS-5 | Non-responsiveness | **0.82** | 0.13 | 0.21 | 0.07 | -0.02 | 0.06 | -0.07 | 0.06 | -0.04 | -0.03 | -0.09 |
|  | SANS-7 | Vocal Inflections | **0.83** | 0.02 | 0.21 | 0.10 | 0.02 | 0.03 | -0.05 | 0.01 | 0.03 | -0.07 | -0.07 |
| Alogia | SANS-9 | Poverty of speech | **0.70** | -0.07 | 0.11 | -0.05 | 0.09 | 0.00 | -0.10 | 0.01 | 0.26 | 0.27 | 0.03 |
|  | SANS-10 | Poverty of speech content | 0.30 | **0.49** | 0.16 | -0.03 | -0.04 | -0.02 | 0.03 | 0.02 | 0.17 | 0.18 | -0.01 |
|  | SANS-11 | Blocking | **0.52** | 0.25 | -0.08 | 0.18 | -0.03 | 0.02 | -0.08 | 0.07 | 0.19 | **0.47** | 0.02 |
|  | SANS-12 | Latency of response | **0.64** | 0.17 | -0.03 | 0.06 | 0.07 | -0.01 | 0.00 | -0.01 | 0.17 | **0.44** | 0.08 |
| Avolition / apathy | SANS-14 | Grooming/hygiene | 0.29 | 0.13 | **0.43** | -0.02 | 0.02 | 0.33 | 0.00 | -0.04 | 0.20 | -0.13 | 0.35 |
|  | SANS-15 | Impersistence | 0.15 | 0.20 | **0.58** | 0.10 | 0.07 | 0.16 | 0.05 | -0.12 | 0.01 | 0.09 | 0.14 |
|  | SANS-16 | Physical anergia | **0.42** | 0.11 | **0.51** | 0.11 | 0.09 | -0.06 | 0.02 | -0.04 | 0.02 | 0.04 | 0.32 |
| Anhedonia / asociality | SANS-18 | Recreational interest/ activity | 0.25 | 0.08 | **0.60** | 0.04 | 0.13 | -0.20 | -0.11 | -0.02 | 0.05 | -0.01 | 0.16 |
|  | SANS-19 | Sexual interest/activity | 0.14 | 0.04 | **0.55** | 0.00 | 0.10 | -0.20 | 0.00 | 0.11 | -0.10 | 0.00 | -0.02 |
|  | SANS-20 | Ability to feel intimacy | 0.21 | 0.15 | **0.65** | 0.09 | -0.02 | -0.03 | -0.14 | 0.06 | 0.14 | -0.12 | -0.21 |
|  | SANS-21 | Relationships | 0.33 | 0.19 | 0.78 | -0.01 | 0.00 | -0.02 | -0.07 | 0.08 | 0.14 | 0.02 | -0.28 |
| Attention | SANS-23 | Social inattentiveness | 0.26 | 0.36 | 0.34 | 0.10 | -0.03 | 0.09 | -0.04 | -0.06 | **0.49** | 0.11 | 0.00 |
|  | SANS-24 | Inattentiveness during testing | 0.35 | **0.44** | 0.26 | 0.01 | -0.03 | 0.11 | -0.03 | 0.01 | **0.42** | 0.07 | 0.06 |
|  |  |  |  |  |  |  |  |  |  |  |  |  |  |

**Supplementary Table 3 Component loadings (rotated) matrix for all 46 items included in the primary first-order exploratory factor analysis using the unweighted least squares extraction method.** Loadings >0.4 in magnitude are shown in full contrast. Note: three items of the original 49 were excluded as they did not meet assumptions of the analysis.

|  |  |  | F1 | F2 | F3 | F4 | F5 | F6 | F7 | F8 | F9 | F10 | F11 |
| --- | --- | --- | --- | --- | --- | --- | --- | --- | --- | --- | --- | --- | --- |
| *% variance explained* |  |  | 19.4 | 9.4 | 9.1 | 5 | 3.8 | 3.4 | 3 | 2.8 | 2.5 | 2.4 | 2.3 |
| Hallucinations | SAPS-H1 | Auditory | 0.01 | -0.08 | 0.09 | 0.23 | **0.71** | -0.07 | -0.04 | 0.15 | -0.02 | 0.09 | 0.06 |
|  | SAPS-H2 | Voices commenting | 0.05 | -0.02 | 0.12 | 0.23 | **0.79** | 0.04 | -0.04 | 0.15 | -0.05 | -0.01 | -0.09 |
|  | SAPS-H3 | Voices conversing | 0.06 | -0.03 | 0.04 | 0.24 | **0.68** | -0.03 | 0.05 | 0.08 | 0.04 | -0.06 | 0.01 |
|  | SAPS-H4 | Somatic or Tactile | -0.10 | 0.01 | 0.01 | 0.21 | 0.25 | -0.16 | 0.14 | 0.31 | 0.06 | -0.08 | 0.02 |
|  | SAPS-H5 | Olfactory | -0.03 | 0.02 | 0.04 | 0.26 | 0.10 | 0.03 | -0.01 | **0.45** | -0.02 | 0.02 | -0.07 |
|  | SAPS-H6 | Visual | 0.00 | 0.02 | 0.00 | 0.13 | 0.20 | -0.04 | 0.02 | **0.56** | -0.02 | 0.01 | -0.03 |
| Delusions | SAPS-D2 | Jealous | -0.03 | -0.05 | 0.01 | 0.16 | 0.01 | 0.05 | -0.04 | 0.06 | 0.00 | -0.02 | -0.34 |
|  | SAPS-D4 | Grandiose | -0.14 | 0.17 | -0.11 | 0.12 | -0.01 | 0.33 | **0.46** | -0.02 | -0.03 | -0.05 | -0.05 |
|  | SAPS-D5 | Religious | -0.06 | 0.02 | -0.07 | 0.15 | -0.01 | 0.06 | **0.71** | 0.05 | -0.02 | -0.01 | 0.08 |
|  | SAPS-D7 | Of reference | -0.02 | 0.00 | 0.10 | **0.40** | 0.16 | -0.11 | 0.15 | -0.09 | 0.01 | 0.05 | -0.18 |
|  | SAPS-D8 | Of being controlled | 0.01 | 0.07 | 0.07 | **0.59** | 0.09 | -0.13 | 0.22 | 0.13 | 0.05 | 0.00 | 0.05 |
|  | SAPS-D9 | Of mind reading | 0.02 | 0.09 | 0.01 | **0.60** | 0.07 | -0.06 | 0.06 | 0.03 | 0.01 | 0.03 | -0.17 |
|  | SAPS-D10 | Thought broadcasting | 0.04 | 0.06 | -0.01 | **0.66** | 0.26 | 0.06 | -0.03 | 0.12 | 0.11 | 0.00 | -0.06 |
|  | SAPS-D11 | Thought insertion | 0.08 | -0.04 | 0.00 | **0.63** | 0.18 | 0.02 | 0.01 | 0.12 | -0.01 | -0.01 | 0.05 |
|  | SAPS-D12 | Thought withdrawal | 0.08 | 0.09 | 0.04 | **0.65** | 0.08 | 0.02 | -0.05 | 0.18 | -0.11 | 0.02 | 0.02 |
| Bizarre behaviour | SAPS-B1 | Appearance | -0.06 | 0.12 | -0.07 | -0.08 | 0.05 | **0.42** | 0.14 | 0.03 | 0.11 | -0.25 | 0.05 |
|  | SAPS-B2 | Social/sexual | 0.05 | 0.17 | -0.05 | 0.02 | -0.07 | **0.42** | 0.20 | 0.06 | -0.01 | 0.02 | -0.02 |
|  | SAPS-B3 | Aggressive/agitated | 0.00 | 0.00 | -0.04 | -0.09 | -0.03 | **0.52** | -0.06 | -0.10 | -0.01 | 0.08 | -0.05 |
|  | SAPS-B4 | Repetitive/stereotyped | 0.14 | 0.15 | 0.05 | 0.21 | -0.09 | 0.03 | 0.02 | 0.17 | 0.06 | 0.01 | -0.02 |
| Pos formal thought disorder | SAPS-P1 | Derailment | 0.05 | **0.84** | 0.04 | 0.05 | -0.01 | -0.05 | 0.03 | -0.01 | 0.08 | 0.02 | -0.01 |
|  | SAPS-P2 | Tangentiality | 0.08 | **0.84** | 0.11 | 0.02 | 0.02 | -0.08 | 0.01 | -0.08 | 0.14 | -0.06 | -0.07 |
|  | SAPS-P3 | Incoherence | 0.10 | **0.43** | 0.06 | 0.09 | 0.01 | 0.18 | -0.04 | 0.02 | 0.15 | -0.17 | 0.04 |
|  | SAPS-P4 | Illogicality | 0.12 | **0.74** | 0.13 | 0.11 | -0.05 | 0.07 | -0.01 | 0.08 | 0.04 | 0.00 | 0.02 |
|  | SAPS-P5 | Circumstantiality | 0.10 | **0.72** | 0.10 | 0.10 | 0.01 | -0.04 | 0.13 | -0.01 | -0.02 | -0.06 | 0.00 |
|  | SAPS-P6 | Pressure of Speech | -0.20 | **0.52** | -0.04 | -0.20 | -0.11 | 0.25 | 0.12 | -0.01 | -0.19 | -0.07 | 0.01 |
|  | SAPS-P7 | Distractible Speech | -0.01 | **0.55** | 0.02 | 0.01 | 0.01 | 0.26 | -0.01 | -0.02 | -0.05 | 0.24 | 0.11 |
|  | SAPS-P8 | Clanging | 0.03 | **0.53** | 0.12 | 0.07 | -0.03 | 0.15 | 0.01 | 0.20 | -0.18 | 0.14 | 0.14 |
| Affective flattening / blunting | SANS-1 | Facial expression | **0.84** | 0.06 | 0.22 | 0.02 | -0.01 | -0.10 | -0.03 | -0.08 | 0.01 | -0.02 | 0.00 |
|  | SANS-2 | Spontaneous movements | **0.81** | 0.10 | 0.13 | 0.08 | 0.03 | -0.08 | 0.04 | -0.05 | -0.12 | -0.06 | 0.11 |
|  | SANS-3 | Expressive gestures | **0.91** | 0.09 | 0.20 | -0.01 | -0.03 | -0.06 | 0.03 | -0.02 | -0.08 | -0.03 | 0.01 |
|  | SANS-4 | Eye contact | **0.50** | 0.06 | 0.16 | 0.00 | 0.03 | 0.12 | -0.06 | 0.02 | 0.16 | 0.01 | 0.08 |
|  | SANS-5 | Non-responsiveness | **0.82** | 0.13 | 0.21 | 0.07 | -0.02 | 0.06 | -0.07 | 0.06 | -0.04 | -0.03 | -0.09 |
|  | SANS-7 | Vocal Inflections | **0.83** | 0.02 | 0.21 | 0.10 | 0.02 | 0.03 | -0.05 | 0.01 | 0.03 | -0.07 | -0.07 |
| Alogia | SANS-9 | Poverty of speech | **0.70** | -0.07 | 0.11 | -0.05 | 0.09 | 0.00 | -0.10 | 0.01 | 0.27 | 0.27 | 0.03 |
|  | SANS-10 | Poverty of speech content | **0.30** | **0.49** | 0.16 | -0.03 | -0.04 | -0.02 | 0.03 | 0.02 | 0.17 | 0.18 | -0.01 |
|  | SANS-11 | Blocking | **0.52** | 0.25 | -0.08 | 0.18 | -0.03 | 0.02 | -0.08 | 0.07 | 0.20 | **0.47** | 0.02 |
|  | SANS-12 | Latency of response | **0.64** | 0.17 | -0.03 | 0.06 | 0.07 | -0.01 | 0.00 | -0.01 | 0.17 | **0.44** | 0.08 |
| Avolition / apathy | SANS-14 | Grooming/hygiene | 0.29 | 0.12 | **0.43** | -0.02 | 0.02 | 0.33 | 0.00 | -0.04 | 0.20 | -0.13 | 0.35 |
|  | SANS-15 | Impersistence | 0.15 | 0.20 | **0.58** | 0.10 | 0.07 | 0.16 | 0.05 | -0.12 | 0.01 | 0.09 | 0.14 |
|  | SANS-16 | Physical anergia | **0.42** | 0.11 | **0.51** | 0.11 | 0.09 | -0.06 | 0.02 | -0.04 | 0.02 | 0.03 | 0.32 |
| Anhedonia / asociality | SANS-18 | Recreational interest/ activity | 0.25 | 0.08 | **0.60** | 0.04 | 0.13 | -0.20 | -0.11 | -0.02 | 0.05 | -0.01 | 0.16 |
|  | SANS-19 | Sexual interest/activity | 0.14 | 0.04 | **0.55** | 0.00 | 0.10 | -0.20 | 0.00 | 0.11 | -0.10 | 0.00 | -0.02 |
|  | SANS-20 | Ability to feel intimacy | 0.21 | 0.15 | **0.65** | 0.09 | -0.02 | -0.03 | -0.14 | 0.06 | 0.14 | -0.12 | -0.21 |
|  | SANS-21 | Relationships | 0.33 | 0.19 | **0.78** | -0.01 | 0.00 | -0.02 | -0.07 | 0.08 | 0.14 | 0.02 | -0.28 |
| Attention | SANS-23 | Social inattentiveness | 0.26 | 0.36 | 0.34 | 0.10 | -0.03 | 0.09 | -0.04 | -0.06 | **0.49** | 0.11 | 0.00 |
|  | SANS-24 | Inattentiveness during testing | 0.35 | **0.44** | 0.26 | 0.01 | -0.03 | 0.11 | -0.03 | 0.01 | **0.42** | 0.07 | 0.06 |
|  |  |  |  |  |  |  |  |  |  |  |  |  |  |

**Supplementary Table 4 Component loadings (rotated) matrix for all 46 items included in the primary first-order exploratory factor analysis using the generalised least squares extraction method.** Loadings >0.4 in magnitude are shown in full contrast. Note: three items of the original 49 were excluded as they did not meet assumptions of the analysis.

|  |  |  | F1 | F2 | F3 | F4 | F5 | F6 | F7 | F8 | F9 | F10 | F11 |
| --- | --- | --- | --- | --- | --- | --- | --- | --- | --- | --- | --- | --- | --- |
| *% variance explained* |  |  | 19.4 | 9.4 | 9.1 | 5 | 3.8 | 3.4 | 3 | 2.8 | 2.5 | 2.4 | 2.3 |
| Hallucinations | SAPS-H1 | Auditory | 0.00 | -0.08 | 0.26 | 0.07 | **0.67** | 0.08 | -0.10 | -0.03 | -0.03 | 0.09 | 0.00 |
|  | SAPS-H2 | Voices commenting | 0.05 | -0.01 | 0.20 | 0.12 | **0.93** | -0.01 | 0.09 | -0.03 | -0.01 | -0.08 | 0.01 |
|  | SAPS-H3 | Voices conversing | 0.07 | -0.01 | 0.26 | -0.01 | **0.64** | -0.03 | -0.10 | 0.04 | 0.03 | 0.11 | -0.02 |
|  | SAPS-H4 | Somatic or Tactile | -0.11 | 0.02 | 0.30 | 0.02 | 0.24 | -0.05 | -0.13 | 0.07 | -0.03 | 0.03 | -0.05 |
|  | SAPS-H5 | Olfactory | -0.04 | 0.01 | 0.36 | 0.09 | 0.13 | 0.01 | 0.08 | -0.02 | 0.00 | -0.10 | 0.04 |
|  | SAPS-H6 | Visual | -0.01 | 0.02 | 0.28 | 0.06 | 0.18 | 0.04 | -0.01 | 0.01 | -0.07 | -0.11 | -0.05 |
| Delusions | SAPS-D2 | Jealous | -0.05 | -0.04 | 0.17 | 0.12 | 0.03 | 0.01 | 0.03 | -0.07 | -0.01 | -0.28 | 0.07 |
|  | SAPS-D4 | Grandiose | -0.13 | 0.19 | 0.14 | -0.14 | -0.05 | -0.07 | 0.29 | 0.36 | 0.01 | -0.04 | 0.05 |
|  | SAPS-D5 | Religious | -0.06 | 0.02 | 0.13 | -0.08 | 0.01 | -0.01 | 0.05 | **0.99** | -0.02 | 0.02 | -0.01 |
|  | SAPS-D7 | Of reference | 0.00 | 0.01 | 0.37 | 0.07 | 0.15 | -0.04 | -0.15 | 0.11 | 0.06 | -0.02 | -0.08 |
|  | SAPS-D8 | Of being controlled | 0.01 | 0.08 | **0.64** | 0.05 | 0.08 | 0.04 | -0.08 | 0.16 | -0.02 | 0.08 | 0.05 |
|  | SAPS-D9 | Of mind reading | 0.04 | 0.09 | **0.59** | 0.04 | 0.09 | -0.01 | -0.05 | 0.06 | 0.06 | -0.11 | -0.07 |
|  | SAPS-D10 | Thought broadcasting | 0.04 | 0.06 | **0.70** | -0.02 | 0.22 | 0.03 | 0.01 | -0.01 | 0.07 | 0.03 | -0.18 |
|  | SAPS-D11 | Thought insertion | 0.07 | -0.04 | **0.64** | -0.03 | 0.23 | 0.04 | 0.05 | 0.01 | 0.06 | 0.03 | 0.16 |
|  | SAPS-D12 | Thought withdrawal | 0.08 | 0.07 | **0.67** | 0.06 | 0.07 | 0.06 | 0.04 | 0.00 | -0.08 | 0.01 | 0.10 |
| Bizarre behaviour | SAPS-B1 | Appearance | -0.06 | 0.13 | -0.06 | -0.11 | 0.02 | -0.15 | 0.39 | 0.11 | 0.06 | 0.07 | -0.06 |
|  | SAPS-B2 | Social/sexual | 0.04 | 0.16 | 0.03 | -0.07 | -0.05 | 0.02 | **0.45** | 0.13 | 0.03 | -0.04 | 0.14 |
|  | SAPS-B3 | Aggressive/agitated | -0.02 | -0.01 | -0.11 | -0.05 | -0.08 | 0.09 | **0.45** | -0.04 | -0.02 | 0.03 | -0.10 |
|  | SAPS-B4 | Repetitive/stereotyped | 0.11 | 0.13 | 0.21 | 0.12 | -0.02 | 0.13 | 0.12 | 0.07 | 0.00 | -0.08 | 0.14 |
| Pos formal thought disorder | SAPS-P1 | Derailment | 0.05 | **0.85** | 0.05 | 0.04 | -0.02 | 0.06 | -0.06 | 0.03 | 0.09 | 0.01 | -0.07 |
|  | SAPS-P2 | Tangentiality | 0.09 | **0.86** | 0.02 | 0.11 | -0.03 | 0.00 | -0.11 | -0.03 | 0.09 | 0.03 | -0.08 |
|  | SAPS-P3 | Incoherence | 0.08 | **0.44** | 0.09 | 0.10 | 0.01 | 0.03 | 0.20 | 0.00 | 0.01 | 0.05 | -0.09 |
|  | SAPS-P4 | Illogicality | 0.11 | **0.73** | 0.12 | 0.14 | -0.02 | 0.06 | 0.09 | 0.00 | 0.06 | 0.04 | 0.09 |
|  | SAPS-P5 | Circumstantiality | 0.10 | **0.73** | 0.10 | 0.10 | 0.02 | -0.02 | 0.01 | 0.09 | 0.02 | 0.01 | 0.14 |
|  | SAPS-P6 | Pressure of Speech | -0.16 | **0.52** | -0.19 | -0.08 | -0.13 | -0.19 | 0.29 | 0.07 | -0.06 | -0.02 | 0.02 |
|  | SAPS-P7 | Distractible Speech | -0.02 | **0.54** | 0.00 | -0.07 | 0.01 | 0.16 | 0.28 | -0.02 | 0.09 | 0.12 | -0.05 |
|  | SAPS-P8 | Clanging | 0.03 | **0.51** | 0.09 | 0.08 | 0.02 | 0.09 | 0.22 | 0.06 | -0.02 | 0.07 | 0.11 |
| Affective flattening / blunting | SANS-1 | Facial expression | **0.85** | 0.06 | -0.01 | 0.16 | -0.01 | 0.06 | -0.14 | -0.01 | 0.12 | 0.09 | 0.10 |
|  | SANS-2 | Spontaneous movements | **0.81** | 0.10 | 0.06 | 0.07 | 0.04 | 0.08 | -0.05 | 0.00 | -0.09 | 0.14 | 0.20 |
|  | SANS-3 | Expressive gestures | **0.92** | 0.09 | -0.02 | 0.15 | -0.01 | 0.06 | -0.05 | 0.01 | 0.02 | 0.05 | 0.20 |
|  | SANS-4 | Eye contact | **0.47** | 0.05 | -0.01 | 0.15 | 0.03 | 0.17 | 0.16 | -0.03 | 0.05 | 0.14 | -0.12 |
|  | SANS-5 | Non-responsiveness | **0.85** | 0.11 | 0.09 | 0.19 | -0.01 | 0.03 | 0.10 | -0.06 | 0.05 | -0.05 | -0.13 |
|  | SANS-7 | Vocal Inflections | **0.87** | 0.01 | 0.11 | 0.18 | 0.04 | 0.00 | 0.08 | -0.06 | 0.08 | -0.03 | -0.19 |
| Alogia | SANS-9 | Poverty of speech | **0.64** | -0.09 | -0.07 | 0.11 | 0.08 | 0.39 | -0.05 | -0.06 | 0.16 | 0.09 | -0.11 |
|  | SANS-10 | Poverty of speech content | 0.28 | **0.48** | -0.05 | 0.16 | -0.01 | 0.24 | 0.01 | 0.01 | 0.15 | 0.01 | -0.08 |
|  | SANS-11 | Blocking | **0.41** | 0.22 | 0.16 | -0.02 | -0.01 | **0.73** | 0.02 | -0.05 | 0.12 | -0.06 | 0.13 |
|  | SANS-12 | Latency of response | **0.57** | 0.15 | 0.03 | -0.04 | 0.04 | **0.60** | -0.02 | 0.02 | 0.06 | 0.11 | -0.17 |
| Avolition / apathy | SANS-14 | Grooming / hygiene | 0.29 | 0.11 | -0.06 | 0.28 | 0.05 | -0.01 | 0.37 | 0.02 | 0.21 | **0.45** | 0.04 |
|  | SANS-15 | Impersistence | 0.17 | 0.20 | 0.06 | **0.41** | 0.07 | -0.01 | 0.13 | -0.02 | 0.10 | **0.48** | 0.00 |
|  | SANS-16 | Physical anergia | **0.43** | 0.12 | 0.09 | 0.34 | 0.10 | 0.06 | -0.05 | 0.02 | 0.06 | **0.53** | 0.01 |
| Anhedonia / asociality | SANS-18 | Recreational interest/ activity | 0.27 | 0.07 | 0.04 | **0.52** | 0.09 | 0.04 | -0.21 | -0.07 | 0.07 | 0.36 | 0.02 |
|  | SANS-19 | Sexual interest/activity | 0.16 | 0.04 | 0.01 | **0.55** | 0.16 | -0.01 | -0.12 | 0.01 | -0.07 | 0.12 | 0.03 |
|  | SANS-20 | Ability to feel intimacy | 0.21 | 0.14 | 0.10 | **0.77** | -0.01 | -0.02 | 0.01 | -0.09 | 0.11 | -0.01 | 0.02 |
|  | SANS-21 | Relationships | 0.34 | 0.19 | 0.01 | **0.79** | 0.03 | 0.02 | 0.00 | -0.05 | 0.18 | 0.03 | -0.05 |
| Attention | SANS-23 | Social inattentiveness | 0.23 | 0.31 | 0.08 | 0.24 | -0.02 | 0.12 | 0.03 | -0.02 | **0.87** | 0.10 | 0.00 |
|  | SANS-24 | Inattentiveness during testing | 0.32 | **0.42** | -0.01 | 0.23 | -0.01 | 0.19 | 0.12 | -0.03 | 0.39 | 0.13 | -0.02 |
|  |  |  |  |  |  |  |  |  |  |  |  |  |  |

**Supplementary Table 5 Component loadings (rotated) matrix for all 46 items included in the primary first-order exploratory factor analysis using the maximum likelihood extraction method.** Loadings >0.4 in magnitude are shown in full contrast. Note: three items of the original 49 were excluded as they did not meet assumptions of the analysis.

|  |  |  | F1 | F2 | F3 | F4 | F5 | F6 | F7 | F8 | F9 | F10 | F11 |
| --- | --- | --- | --- | --- | --- | --- | --- | --- | --- | --- | --- | --- | --- |
| *% variance explained* |  |  | 19.4 | 9.4 | 9.1 | 5 | 3.8 | 3.4 | 3 | 2.8 | 2.5 | 2.4 | 2.3 |
| Hallucinations | SAPS-H1 | Auditory | 0.00 | -0.09 | 0.10 | 0.29 | **0.69** | -0.09 | -0.04 | 0.08 | -0.03 | 0.04 | 0.00 |
|  | SAPS-H2 | Voices commenting | 0.05 | -0.02 | 0.11 | 0.28 | **0.83** | 0.06 | -0.04 | -0.01 | -0.04 | -0.10 | 0.06 |
|  | SAPS-H3 | Voices conversing | 0.07 | -0.02 | 0.03 | 0.28 | **0.66** | -0.04 | 0.03 | -0.05 | 0.08 | 0.06 | -0.06 |
|  | SAPS-H4 | Somatic or Tactile | -0.11 | 0.02 | 0.01 | 0.29 | 0.25 | -0.12 | 0.07 | -0.05 | 0.00 | 0.03 | 0.00 |
|  | SAPS-H5 | Olfactory | -0.03 | 0.02 | 0.05 | 0.35 | 0.12 | 0.04 | -0.03 | 0.02 | -0.10 | -0.09 | 0.10 |
|  | SAPS-H6 | Visual | -0.01 | 0.03 | 0.01 | 0.27 | 0.20 | -0.05 | 0.01 | 0.04 | -0.12 | -0.07 | 0.17 |
| Delusions | SAPS-D2 | Jealous | -0.04 | -0.04 | 0.00 | 0.17 | 0.01 | 0.01 | -0.05 | 0.01 | -0.01 | -0.33 | 0.00 |
|  | SAPS-D4 | Grandiose | -0.14 | 0.18 | -0.13 | 0.14 | -0.04 | 0.34 | 0.34 | -0.06 | -0.03 | -0.03 | -0.06 |
|  | SAPS-D5 | Religious | -0.06 | 0.01 | -0.07 | 0.17 | 0.00 | 0.11 | **0.97** | -0.01 | -0.01 | 0.11 | 0.02 |
|  | SAPS-D7 | Of reference | 0.00 | 0.00 | 0.05 | 0.39 | 0.12 | -0.13 | 0.10 | -0.05 | 0.13 | -0.04 | 0.02 |
|  | SAPS-D8 | Of being controlled | 0.01 | 0.08 | 0.05 | **0.62** | 0.06 | -0.07 | 0.14 | 0.03 | 0.00 | 0.04 | -0.10 |
|  | SAPS-D9 | Of mind reading | 0.04 | 0.09 | -0.01 | **0.60** | 0.04 | -0.06 | 0.04 | -0.01 | 0.07 | -0.08 | 0.07 |
|  | SAPS-D10 | Thought broadcasting | 0.05 | 0.06 | -0.02 | **0.70** | 0.20 | 0.03 | -0.05 | -0.01 | 0.18 | 0.04 | 0.07 |
|  | SAPS-D11 | Thought insertion | 0.08 | -0.04 | 0.01 | **0.64** | 0.18 | 0.06 | -0.02 | 0.06 | -0.04 | -0.02 | -0.15 |
|  | SAPS-D12 | Thought withdrawal | 0.08 | 0.07 | 0.04 | **0.66** | 0.05 | 0.01 | -0.02 | 0.06 | -0.12 | -0.04 | -0.06 |
| Bizarre behaviour | SAPS-B1 | Appearance | -0.06 | 0.13 | -0.06 | -0.06 | 0.04 | **0.43** | 0.09 | -0.14 | 0.06 | 0.06 | -0.01 |
|  | SAPS-B2 | Social/sexual | 0.05 | 0.17 | -0.05 | 0.03 | -0.05 | **0.46** | 0.11 | 0.04 | -0.06 | -0.09 | -0.06 |
|  | SAPS-B3 | Aggressive/agitated | -0.01 | 0.00 | -0.04 | -0.11 | -0.06 | **0.43** | -0.06 | 0.08 | 0.02 | -0.01 | 0.07 |
|  | SAPS-B4 | Repetitive/stereotyped | 0.11 | 0.13 | 0.08 | 0.21 | -0.05 | 0.08 | 0.06 | 0.13 | -0.06 | -0.10 | -0.03 |
| Pos formal thought disorder | SAPS-P1 | Derailment | 0.04 | **0.85** | 0.04 | 0.06 | -0.03 | -0.06 | 0.03 | 0.04 | 0.06 | 0.04 | 0.04 |
|  | SAPS-P2 | Tangentiality | 0.08 | **0.85** | 0.10 | 0.02 | -0.02 | -0.09 | -0.02 | -0.04 | 0.16 | -0.03 | -0.04 |
|  | SAPS-P3 | Incoherence | 0.08 | **0.43** | 0.09 | 0.09 | 0.02 | 0.19 | -0.01 | -0.01 | 0.09 | 0.00 | -0.03 |
|  | SAPS-P4 | Illogicality | 0.10 | **0.72** | 0.15 | 0.11 | -0.03 | 0.07 | 0.00 | 0.05 | 0.00 | -0.02 | -0.08 |
|  | SAPS-P5 | Circumstantiality | 0.09 | **0.72** | 0.10 | 0.10 | 0.02 | 0.01 | 0.10 | -0.04 | -0.04 | -0.06 | -0.14 |
|  | SAPS-P6 | Pressure of Speech | -0.18 | **0.52** | -0.06 | -0.18 | -0.11 | 0.25 | 0.06 | -0.17 | -0.19 | 0.02 | 0.09 |
|  | SAPS-P7 | Distractible Speech | -0.01 | **0.55** | 0.00 | 0.01 | 0.01 | 0.24 | -0.05 | 0.15 | -0.08 | 0.15 | 0.11 |
|  | SAPS-P8 | Clanging | 0.02 | **0.51** | 0.12 | 0.10 | 0.01 | 0.15 | 0.04 | 0.10 | -0.26 | 0.08 | 0.10 |
| Affective flattening / blunting | SANS-1 | Facial expression | **0.84** | 0.07 | 0.22 | -0.01 | -0.02 | -0.12 | 0.00 | 0.03 | 0.07 | -0.01 | -0.13 |
|  | SANS-2 | Spontaneous movements | **0.80** | 0.10 | 0.13 | 0.06 | 0.03 | -0.07 | 0.01 | 0.04 | -0.16 | 0.06 | -0.23 |
|  | SANS-3 | Expressive gestures | **0.91** | 0.10 | 0.20 | -0.02 | -0.02 | -0.05 | 0.03 | 0.04 | -0.07 | -0.04 | -0.15 |
|  | SANS-4 | Eye contact | **0.48** | 0.06 | 0.20 | -0.01 | 0.03 | 0.14 | -0.04 | 0.12 | 0.07 | 0.10 | 0.07 |
|  | SANS-5 | Non-responsiveness | **0.85** | 0.13 | 0.19 | 0.10 | -0.03 | 0.03 | -0.06 | -0.03 | -0.03 | -0.04 | 0.20 |
|  | SANS-7 | Vocal Inflections | **0.86** | 0.03 | 0.19 | 0.12 | 0.01 | 0.04 | -0.06 | -0.05 | 0.06 | -0.03 | 0.15 |
| Alogia | SANS-9 | Poverty of speech | **0.67** | -0.07 | 0.15 | -0.06 | 0.09 | -0.03 | -0.06 | 0.34 | 0.22 | 0.04 | 0.04 |
|  | SANS-10 | Poverty of speech content | 0.29 | **0.50** | 0.16 | -0.03 | -0.02 | -0.01 | 0.01 | 0.21 | 0.12 | 0.00 | 0.10 |
|  | SANS-11 | Blocking | **0.45** | 0.26 | -0.04 | 0.16 | -0.02 | -0.01 | -0.06 | **0.69** | 0.02 | -0.06 | -0.04 |
|  | SANS-12 | Latency of response | **0.60** | 0.17 | 0.00 | 0.04 | 0.05 | -0.04 | 0.01 | **0.50** | 0.09 | 0.12 | 0.09 |
| Avolition / apathy | SANS-14 | Grooming/hygiene | 0.28 | 0.12 | **0.46** | -0.05 | 0.03 | 0.37 | -0.02 | 0.01 | 0.10 | 0.24 | -0.11 |
|  | SANS-15 | Impersistence | 0.15 | 0.21 | **0.55** | 0.07 | 0.04 | 0.12 | -0.04 | 0.00 | 0.04 | 0.22 | -0.06 |
|  | SANS-16 | Physical anergia | **0.42** | 0.12 | **0.52** | 0.10 | 0.08 | -0.06 | 0.00 | 0.06 | -0.03 | 0.38 | -0.07 |
| Anhedonia / asociality | SANS-18 | Recreational interest/ activity | 0.25 | 0.08 | **0.60** | 0.04 | 0.10 | -0.23 | -0.06 | 0.03 | 0.01 | 0.14 | -0.05 |
|  | SANS-19 | Sexual interest/activity | 0.14 | 0.04 | **0.54** | 0.03 | 0.13 | -0.20 | 0.02 | -0.02 | -0.16 | -0.04 | 0.07 |
|  | SANS-20 | Ability to feel intimacy | 0.20 | 0.15 | **0.69** | 0.10 | -0.02 | -0.04 | -0.07 | -0.03 | 0.10 | -0.27 | 0.01 |
|  | SANS-21 | Relationships | 0.32 | 0.21 | **0.77** | 0.02 | 0.02 | -0.05 | -0.02 | 0.02 | 0.12 | -0.25 | 0.12 |
| Attention | SANS-23 | Social inattentiveness | 0.25 | 0.36 | 0.36 | 0.08 | -0.05 | 0.13 | -0.05 | 0.19 | **0.47** | 0.02 | 0.00 |
|  | SANS-24 | Inattentiveness during testing | 0.33 | **0.44** | 0.31 | 0.00 | -0.02 | 0.16 | -0.04 | 0.19 | 0.29 | 0.04 | -0.02 |
|  |  |  |  |  |  |  |  |  |  |  |  |  |  |

**Supplementary Table 6 First order principal component analysis component loadings (structure) matrix – analysis rerun without items relating to the Attention subscale.** Loadings >0.4 in magnitude are shown in bold.

|  |  |  | F1 | F2 | F3 | F4 | F5 | F6 | F7 | F8 | F9 | F10 | F11 |
| --- | --- | --- | --- | --- | --- | --- | --- | --- | --- | --- | --- | --- | --- |
| *% variance explained* |  |  | 18.7 | 9.7 | 9.6 | 5.2 | 4 | 3.5 | 3.1 | 2.8 | 2.6 | 2.4 | 2.3 |
| Hallucinations | SAPS-H1 | Auditory | 0.03 | -0.08 | 0.30 | -0.11 | -0.07 | **-0.82** | 0.04 | 0.21 | -0.07 | 0.10 | -0.03 |
|  | SAPS-H2 | Voices commenting | 0.06 | -0.01 | 0.29 | -0.16 | -0.03 | **-0.85** | 0.01 | 0.21 | 0.11 | -0.03 | 0.05 |
|  | SAPS-H3 | Voices conversing | 0.08 | -0.02 | 0.29 | -0.09 | -0.09 | **-0.82** | -0.07 | 0.13 | 0.06 | -0.04 | -0.02 |
|  | SAPS-H4 | Somatic or tactile | -0.11 | 0.00 | 0.17 | -0.04 | -0.31 | **-0.41** | -0.23 | **0.41** | 0.12 | 0.03 | 0.17 |
|  | SAPS-H5 | Olfactory | -0.02 | 0.02 | 0.29 | -0.05 | 0.04 | -0.16 | -0.03 | **0.73** | 0.10 | 0.05 | 0.09 |
|  | SAPS-H6 | Visual | 0.02 | 0.03 | 0.15 | 0.00 | -0.08 | -0.29 | -0.04 | **0.78** | 0.01 | 0.02 | 0.02 |
| Delusions | SAPS-D2 | Jealous | -0.03 | -0.05 | 0.12 | -0.01 | 0.06 | -0.06 | 0.02 | 0.09 | **0.81** | -0.02 | 0.12 |
|  | SAPS-D4 | Grandiose | -0.15 | 0.20 | 0.12 | 0.14 | 0.28 | 0.02 | **-0.68** | 0.05 | 0.06 | -0.18 | -0.08 |
|  | SAPS-D5 | Religious | -0.08 | 0.03 | 0.14 | 0.08 | -0.07 | -0.04 | **-0.80** | 0.03 | -0.06 | -0.01 | 0.07 |
|  | SAPS-D7 | Of reference | 0.01 | 0.05 | **0.48** | -0.14 | -0.06 | -0.26 | -0.22 | -0.07 | 0.34 | 0.28 | -0.16 |
|  | SAPS-D8 | Of being controlled | 0.03 | 0.09 | **0.64** | -0.13 | -0.19 | -0.26 | -0.32 | 0.16 | 0.07 | 0.15 | 0.18 |
|  | SAPS-D9 | Of mind reading | 0.05 | 0.13 | **0.70** | -0.04 | -0.08 | -0.18 | -0.12 | 0.09 | 0.24 | 0.10 | 0.03 |
|  | SAPS-D10 | Thought broadcasting | 0.08 | 0.10 | **0.75** | -0.03 | 0.06 | **-0.42** | -0.04 | 0.22 | 0.09 | 0.00 | 0.02 |
|  | SAPS-D11 | Thought insertion | 0.09 | -0.04 | **0.70** | -0.04 | -0.05 | **-0.35** | -0.11 | 0.17 | 0.01 | -0.01 | 0.19 |
|  | SAPS-D12 | Thought withdrawal | 0.11 | 0.11 | **0.74** | -0.09 | 0.01 | -0.21 | -0.03 | 0.28 | 0.00 | 0.00 | 0.14 |
| Bizarre behaviour | SAPS-B1 | Appearance | -0.08 | 0.11 | -0.12 | 0.09 | 0.28 | -0.06 | -0.30 | -0.01 | 0.00 | **-0.66** | 0.11 |
|  | SAPS-B2 | Social/sexual | 0.04 | 0.17 | -0.02 | 0.03 | **0.52** | 0.08 | **-0.43** | 0.13 | 0.06 | -0.15 | 0.16 |
|  | SAPS-B3 | Aggressive/agitated | -0.02 | 0.00 | -0.08 | 0.08 | **0.76** | 0.09 | 0.01 | -0.06 | 0.01 | -0.17 | -0.09 |
|  | SAPS-B4 | Repetitive/stereotyped | 0.12 | 0.13 | 0.22 | -0.10 | -0.10 | 0.02 | -0.04 | 0.10 | 0.14 | -0.14 | **0.76** |
| Formal thought disorder | SAPS-P1 | Derailment | 0.11 | **0.87** | 0.10 | -0.14 | 0.08 | 0.02 | -0.06 | 0.02 | -0.05 | -0.03 | 0.16 |
|  | SAPS-P2 | Tangentiality | 0.15 | **0.86** | 0.06 | -0.22 | 0.05 | 0.00 | -0.03 | -0.06 | 0.02 | -0.07 | 0.15 |
|  | SAPS-P3 | Incoherence | 0.14 | 0.48 | 0.18 | -0.11 | 0.13 | -0.03 | 0.07 | -0.06 | -0.05 | **-0.51** | 0.22 |
|  | SAPS-P4 | Illogicality | 0.19 | **0.79** | 0.18 | -0.23 | 0.16 | 0.04 | -0.03 | 0.12 | -0.09 | -0.10 | 0.16 |
|  | SAPS-P5 | Circumstantiality | 0.15 | **0.78** | 0.15 | -0.20 | 0.01 | -0.01 | -0.19 | 0.04 | -0.04 | -0.04 | 0.08 |
|  | SAPS-P6 | Pressure of Speech | -0.23 | **0.54** | -0.23 | 0.05 | 0.22 | 0.21 | -0.26 | 0.03 | -0.08 | -0.24 | 0.07 |
|  | SAPS-P7 | Distractible Speech | 0.00 | **0.59** | -0.01 | -0.06 | **0.43** | -0.03 | -0.11 | -0.01 | -0.20 | 0.11 | 0.35 |
|  | SAPS-P8 | Clanging | 0.05 | **0.57** | 0.11 | -0.17 | 0.22 | 0.03 | -0.09 | 0.28 | -0.29 | 0.00 | 0.26 |
| Affective flattening/blunting | SANS-1 | Facial expression | **0.88** | 0.12 | 0.07 | -0.39 | -0.03 | -0.03 | 0.15 | -0.10 | -0.09 | 0.05 | 0.11 |
|  | SANS-2 | Spontaneous movements | **0.83** | 0.13 | 0.12 | -0.30 | -0.05 | -0.08 | 0.03 | -0.07 | -0.12 | 0.01 | 0.16 |
|  | SANS-3 | Expressive gestures | **0.91** | 0.14 | 0.05 | -0.38 | 0.00 | -0.02 | 0.08 | -0.06 | -0.09 | 0.04 | 0.16 |
|  | SANS-4 | Eye contact | **0.57** | 0.07 | 0.00 | -0.28 | 0.16 | -0.09 | 0.11 | -0.08 | -0.07 | -0.10 | 0.34 |
|  | SANS-5 | Non-responsiveness | **0.86** | 0.18 | 0.12 | -0.37 | 0.09 | -0.03 | 0.13 | 0.04 | -0.02 | -0.01 | 0.16 |
|  | SANS-7 | Vocal Inflections | **0.87** | 0.08 | 0.16 | -0.37 | 0.04 | -0.08 | 0.13 | -0.02 | -0.03 | -0.03 | 0.11 |
| Alogia | SANS-9 | Poverty of speech | **0.76** | -0.02 | 0.00 | -0.23 | 0.15 | -0.16 | 0.25 | -0.10 | -0.13 | 0.21 | 0.27 |
|  | SANS-10 | Poverty of speech content | **0.38** | 0.55 | -0.03 | -0.27 | 0.08 | -0.01 | 0.01 | -0.07 | -0.07 | 0.15 | **0.42** |
|  | SANS-11 | Blocking | **0.58** | 0.30 | 0.20 | -0.03 | 0.23 | -0.09 | 0.15 | -0.03 | -0.09 | 0.34 | **0.49** |
|  | SANS-12 | Latency of response | **0.69** | 0.23 | 0.09 | -0.09 | 0.20 | -0.18 | 0.09 | -0.11 | -0.14 | 0.34 | **0.40** |
| Avolition/apathy | SANS-14 | Grooming/hygiene | 0.35 | 0.14 | 0.00 | **-0.53** | 0.28 | -0.06 | -0.03 | -0.12 | -0.37 | -0.34 | 0.26 |
|  | SANS-15 | Impersistence | 0.23 | 0.24 | 0.10 | **-0.70** | 0.28 | -0.11 | -0.10 | -0.08 | -0.14 | 0.09 | 0.10 |
|  | SANS-16 | Physical anergia | 0.52 | 0.15 | 0.14 | **-0.65** | -0.01 | -0.17 | 0.02 | -0.06 | -0.31 | 0.06 | 0.18 |
| Anhedonia/asociality | SANS-18 | Recreational interest/ activity | 0.36 | 0.11 | 0.09 | **-0.72** | -0.17 | -0.19 | 0.22 | -0.06 | -0.18 | 0.04 | 0.05 |
|  | SANS-19 | Sexual interest/activity | 0.20 | 0.07 | 0.01 | **-0.66** | -0.24 | -0.17 | 0.05 | 0.12 | 0.06 | 0.09 | 0.08 |
|  | SANS-20 | Ability to feel intimacy | 0.33 | 0.20 | 0.14 | **-0.74** | -0.05 | -0.02 | 0.23 | 0.04 | 0.20 | -0.14 | 0.07 |
|  | SANS-21 | Relationships | 0.47 | 0.27 | 0.06 | -0.80 | 0.02 | -0.05 | 0.19 | 0.05 | 0.14 | -0.02 | 0.09 |
|  |  |  |  |  |  |  |  |  |  |  |  |  |  |

**Supplementary Table 7 Component loadings (rotated matrix) for all 11 variables included in the second-order principal component analysis – analysis rerun without Attention subscale.** The coefficients of components 4, 6, 7 and 10 have been inverted to aid interpretation. This is appropriate, since in the first-order PCA the loadings associated with these four components were negative, i.e. of a different sign to the other components. Loadings >0.4 in magnitude are shown in bold.

|  | NEG | POS | DIS |
| --- | --- | --- | --- |
| *% variance explained* | 15.5 | 13.4 | 12 |
| (1) Negative Symptoms | **0.79** | -0.04 | -0.04 |
| (2) Thought Disorder | 0.34 | 0.08 | **0.59** |
| (3) Delusions | 0.16 | **0.71** | -0.02 |
| (4) Social dysfunction | **0.67** | 0.09 | -0.05 |
| (5) Bizarre behaviour | 0.05 | -0.19 | **0.61** |
| (6) Auditory hallucinations | 0.19 | **0.64** | -0.17 |
| (7) Grandiose delusions | -0.33 | 0.33 | **0.49** |
| (8) Other hallucinations | -0.11 | **0.57** | 0.10 |
| (9) Jealous delusions | -0.24 | 0.29 | -0.20 |
| (10) Appearance/ incoherence | -0.15 | -0.09 | **0.41** |
| (11) Alogia | **0.43** | 0.14 | 0.39 |
|  |  |  |  |
